# Ras isoforms: signaling specificities in CD40 pathway

**DOI:** 10.1186/s12964-019-0497-1

**Published:** 2020-01-06

**Authors:** Arathi Nair, Sushmita Chakraborty, Late Anirban Banerji, Ankita Srivastava, Charudutta Navare, Bhaskar Saha

**Affiliations:** 1 National Centre for Cell Science, Ganeshkhind, Pune, 411007 India; 2 Department of Transplant Immunology and Immunogenetics, All India Institute of Medical Sciences, New Delhi, 1100029 India; 3 Bioinformatics Centre, University of Pune, Pune, 411007 India

**Keywords:** Ras isoforms, CD40, Signal transduction, Specificity

## Abstract

**Background:**

Ras are small cellular GTPases which regulate diverse cellular processes. It has three isoforms: H-Ras, K-Ras, and N-Ras. Owing to the N-terminus (1–165 residues) sequence homology these isoforms were thought to be functionally redundant. However, only K-Ras-deficient mice but not H-Ras- and N-Ras-deficient mice show embryonic lethality. Similarly, mutations in a given Ras isoform are associated with a particular type of cancer. Moreover, we have previously reported that Ras isoforms perform unique functions in *Leishmania major* infection. Thus, Ras isoforms are implicated to have signaling and functional specificity but the mechanism remains to be elucidated*.*

**Result:**

Using CD40 as a model receptor, we showed that depending on the strength of signaling, specific Ras isoforms are activated. Weak CD40 signal activates N-Ras, whereas strong signal activates H-Ras and K-Ras. Additionally, we showed that suppression of N-Ras expression reduced CD40-induced extracellular signal–regulated kinase-1/2 (ERK-1/2) activation and Interleukin (IL)-10 production; whereas suppression of H-Ras or K-Ras reduced CD40-induced p38 mitogen-activated protein kinase (p38MAPK) activation and IL-12 production. Furthermore, we showed that Ras isoforms have activator (GEF) specificity as weak CD40 signal-activated N-Ras requires Sos-1/2 whereas strong CD40 signal-activated H-Ras/K-Ras requires Ras-GRP as the guanine-nucleotide exchange factor (GEF) inducing ERK-1/2- or p38MAPK-mediated IL-10 or IL-12 productions, respectively, in macrophages. Silencing of syk reduced CD40-induced N-Ras activation but silencing of lyn inhibited H-Ras and K-Ras activation. In CD40 signaling, Ras isoforms also showed effector specificity; while H-Ras and K-Ras showed specificity for phosphatidyl inositol-3 kinase activation at high dose of CD40 stimulation, N-Ras primarily associated with Raf-1 at low dose of CD40 stimulation. Moreover, fractal analysis showed that functional site surface roughness for H-Ras (SurfaceFD = 2.39) and K-Ras (SurfaceFD = 2.39) are similar but significantly different from N-Ras (SurfaceFD = 2.25).

**Conclusion:**

The activator and effector specificities of Ras isoforms in CD40 signaling indicated their differential involvement in CD40 pathway and in maintaining the reciprocity. Our observations reveal Ras-regulated signaling outcome and its potential for developing Ras isoform-targeted immunotherapy and prophylaxis.

**Graphical abstract:**

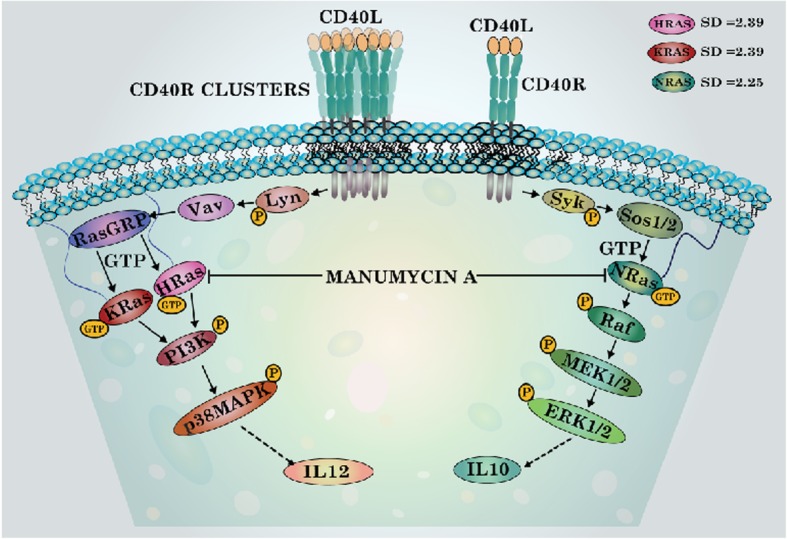

## Background

Small cellular GTPases Ras are known as ‘molecular switch’ of the cell and have established roles in various cellular processes such as proliferation, apoptosis, oncogenesis and Ras-related developmental defects called Rasopathies [1]. This ubiquitously expressed 21 kDa protein has three isoforms namely –H-Ras, K-Ras (K-Ras 4A and K-Ras 4B) and N-Ras. The primary structure of the isoforms has 82–90% similarity in the amino acid sequence, and structural variability lies in the C- terminus Hyper Variable Region (HVR), that undergoes a series of enzymatic post-translational modications resulting in dual palmitoylation in H-Ras (Residue 181, 184), single palmitoylation in N-Ras (Residue 181) and a polylysine stretch in K-Ras [2]. Owing to the high sequence homology in their N-terminal 1–165 amino acid residues, wherein the effector binding domain lies, Ras isoforms- H-Ras, K-Ras, N-Ras- were previously considered to be functionally redundant. However, several observations imply that Ras isoforms may not be as redundant as they were thought to be. Firstly, K-Ras mutation is more frequently observed in pancreatic and colon cancers while N-Ras mutation is observed in acute leukemia [3]. Although these observations associate mutated Ras isoforms with particular tissues only during metastatic transformation, one might imply these isoforms in non-transformed tissue-specific functions. However, that remains to be demonstrated. Secondly, K-Ras deficiency is embryonically lethal [4] but H-Ras or N-Ras deficient mice show no developmental abnormality [5] suggesting that K-Ras is indispensable in embryonic development. Thirdly, K-Ras has higher tumor-inducing potential compared to H-Ras or N-Ras [6], implying differential contribution of the isoforms towards cellular activation or proliferation. Fourthly, germ-line mutational studies suggest that H-Ras mutation is observed in more than 86% in Costello syndrome cases [7], whereas K-Ras mutation is frequently observed in cardio-facial-cutaneous (CFC) syndrome [8], suggesting isoform specific association with these syndromes. These observations describe differential involvements of Ras isoforms in oncogenesis or embryonic development and imply functional non-redundancy in Ras isoforms. Previous study from our group has demonstrated that H-Ras, K-Ras and N-Ras have differential involvements in *Leishmania major* infection and inhibition of N-Ras reduced *L. major* infection in macrophages [9]. This finding reinstates the functional specificty of Ras isoforms in a disease model. As the catalytic domain in Ras protein lies in the conserved G-domain, the differences in their functions and any signaling specificity remains a paradox.

CD40, a trans-membrane costimulatory receptor expressed on antigen-presenting cells such as macrophages and dendritic cells, plays crucial roles in autoimmune and infectious diseases, transplant rejection and tumor regression [10]. Blockade of CD40-CD154 interaction prevents autoimmune diseases and transplant rejection but abrogates host-protection against pathogens. Macrophage expressed CD40, induces activation of extracellular signal–regulated kinase-1/2 (ERK-1/2)-mediated anti-inflammatory IL-10 production and p38 mitogen-activated protein kinase (p38MAPK)-mediated pro-inflammatory IL-12 production, depending on the strength of signaling [11] reflecting a functional duality for CD40 [12].

It was reported that a generic dominant negative mutant of Ras inhibited CD40 signaling in B cells [13] and endothelial cells [14]. But, the possibility of differential involvement of the Ras isoforms in CD40 mediated counteractive signaling in macrophages was never proposed. Using reciprocal CD40 signaling in macrophages [11] as a model physiological function, we examined the differential activation and function of Ras isoforms. Our results demonstrate CD40-dose-dependent differential activation of Ras isoforms. For their activation, the Ras isoforms require different guanine nucleotide exchange factors (GEFs). As effector molecules, Phosphatidyl inositol-3 kinase (PI3K) and Rapidly Accelerated Fibrosarcoma (Raf-1), both containing Ras-binding domain (RBD), were differentially activated by Ras isoforms in CD40 pathway. Our observations suggest that Ras isoforms are differentially involved in CD40 pathway depending upon the strength of CD40 signaling. We also performed the fractal analysis of Ras isoforms as the Fractal dimension (FD) or surface roughness quantification is an important tool in understanding the structural and functional properties of a protein [15, 16]. Present study shows that Ras isoforms have activator and effector specificities and their fractal dimensions are different.

Thus, although Ras isoforms have so far been thought as structurally and functionally similar, we demonstrated in this study that Ras isoforms do have unique activation requirements, effector specificities and functions in non-transformed cells.

## Methods

### Animals and culture of cell lines

BALB/c (Jackson Laboratories, Bar Harbor, ME) were originally obtained from Jackson Laboratories (Bar Harbor, Maine, USA). The animals were subsequently bred and maintained in the institute’s experimental facility in Thoren Caging systems (Philadelphia, PA, USA). Studies were perfomed using mice of the age group 6–8 weeks. All experimentations were performed in accordance with the protocol approved by the Institutional Animal Care and Use Committee (IACUC) and the Committee for the Purpose of Control and Supervision of Experiments on Animals (CPCSEA), the regulatory authorities for animal experimentation.

A mouse macrophage cell line, P388D1 was procured from ATCC (American Type Culture Collection). The cells were cultured in RPMI-1640 medium containing penicillin (70 μg/ml), streptomycin (100 μg/ml), 2-mercaptoethanol (50 μM), sodium pyruvate (1 μM), HEPES (20 μM) and 10% heat inactivated Fetal bovine Serum (FBS) (GIBCO, BRL) in a humidified atmosphere of 5% CO_2_ at 37 °C CO_2_ incubator (Forma scientific). This cell line was used for in-vitro transfection studies.

### Isolation of peritoneal macrophages

BALB/c mice were intraperitoneally injected with 2 ml of 3% thioglycolate. After 4 days the mice were sacrificed and peritoneal exudates were harvested by 18-gauge needle in sterile Hanks Balanced Salt Solution (HBSS). The peritoneal exudates were subjected to centrifugation at 1200 rpm for 8 min and the pellet was resuspended in RPMI-1640 supplemented with heat inactivated FBS. The cells were counted in Neubauer’s counting chamber. The cells were plated according to the requirement of the experiment. After 12 h of rest the old media was replaced with fresh media for the removal of non-adherent cells and further cultured in RPMI 1640 with 10% heat inactivated FBS, in a humidified atmosphere of 5% CO_2_ at 37 °C CO_2_ incubator (Forma scientific).

### Immunoblotting and antibodies

4 × 10^6^ cells were plated for western blot analysis. After stimulation, the cells were washed twice with ice-cold Phosphate buffered saline (PBS) and resuspended in appropriate (~ 200-300 μl) amount of ice-cold lysis buffer for 30 min and kept for gentle rocking at 4 °C. Cell lysates were centrifuged at 14,000×g for 15 min at 4 °C. The supernatant was transferred to fresh microcentrifuge tube. Protein content of the sample was measured using Pierce Bicinchoninic acid assay (BCA) protein assay kit or Bradford’s assay and samples were stored at − 70 °C for further use. Equal concentration of samples in Laemmli sample buffer were loaded on SDS-polyacrylamide. Electrophoresis was carried out at a constant voltage of 60 V. The proteins from polyacrylamide gel were transferred to PVDF (activated with methanol for few seconds) or nitrocellulose membrane at 180 mA for 3.5 h using a trans-blot system. The transfer was checked with Ponceau S solution. The membrane was washed with Tris-buffered saline, 0.1% Tween 20 (TBS-T) to remove all traces of Ponceau S and blocked with blocking buffer (5% BSA in TBS-T buffer) for 1 h at room temperature. The blocked membrane was washed thrice with TBS-T for 10 min each. Blots were then incubated with primary antibody (dilution 1:1000) for 3 h at room temperature or overnight at 4 °C followed by washing three times. Blots were then incubated with secondary antibody conjugated to HRP (dilution 1:2000 or 3000) for 1 h. The blots were washed three times with TBS-T and the immuno-reactive bands were visualized by the ECL substrate in dark room. For sequential detection, membranes were stripped and re-probed with other antibodies. The membranes were stripped using stripping buffer for 2 h at room temperature with rocking. The following antibodies were used – H Ras (Santa Cruz Biotechnology- Sc29), K Ras (Santa Cruz Biotechnology - Sc30), N Ras (Santa Cruz Biotechnology - Sc31), phospho-p38 (Santa Cruz Biotechnology -Sc7973), p38 (Santa Cruz Biotechnology -Sc7972), ERK1/2 (Santa Cruz Biotechnology -Sc154), phospho-ERK (Santa Cruz Biotechnology -Sc7383), β-actin (Santa Cruz Biotechnology -Sc1616), phosho-tyrosine, Vav (Santa Cruz Biotechnology -Sc132), Sos ½ (Santa Cruz Biotechnology -Sc259), Ras-GRP (Santa Cruz Biotechnology -Sc8430), CD71 (Santa Cruz Biotechnology -Sc9099), phospho-Lyn (Cell signaling- 2731 ), Lyn (Cell signalling Technology-2732), phospho Syk (Cell signaling-2711 ), Syk (Cell signaling-2712), CD40 (Santa Cruz Biotechnology - Sc9096), phospho-PI3K (Cell signaling-4228), PI3K (Santacruz-Sc7174), phospho-Raf (Cell signalling Technology-9121 s), Raf (Cell signalling Technology-9427 ), Anti Ras (Cell signalling Technology – 3339).

### Co-immunoprecipitation

5 × 10^6^ macrophages were plated. After stimulation, cells were washed with chilled PBS and lysed with lysis buffer containing NP-40. Cell lysates were centrifuged at 14,000 g for 10 min at 4 °C. After centrifugation, pellet was discarded and supernatant was transferred to a new microcentrifuge. The protein concentration was estimated from supernatant using BCA or Bradfords assay. 200 μg of protein was mixed with 1 μg of antibody and 15 μl of protein G agarose beads, followed by overnight incubation at 4 °C with mild rocking. The antibody bound complexes were washed 4 times with lysis buffer followed by resuspension in the Laemmli sample buffer. The Laemmli sample buffer containing the complexes was heated at 95°C for 5–10 min, followed by electrophoresis and western blotting.

### Active Ras-pull down assay

10 million cells were used for Active-Ras Pull-Down Assay. After stimulation with indicated doses of anti-CD40 (NA/LE BD Pharmingen) or CD40 ligand (Alexis), cells were gently rinsed with ice cold PBS at least thrice. After removal of PBS, 600 μl of lysis buffer was added and the plates were either rocked at 4 °C or cells were scraped. The cells were then centrifuged at 16,000 g for 15 min at 4 °C. The supernatants were transferred to fresh microcentrifuge tube. The protein concentration was estimated using BCA kit (Pierce) or Bradfords assay (Biorad). For each sample a spin cup was placed into a collection tube and 1–2 glutathione resin cubes were added to each spin cup, followed by 80 μg of GST-Raf1 RBD. Following this 500- 700 μg of protein was loaded onto the spin cup and the collection tubes were sealed with laboratory film to prevent leakage. The tubes were incubated at 4 °C for 2–3 h with gentle rocking. After incubation, the spin cups with collection tubes were centrifuged at 6000 g for 1 min. The spin cup (with resin) was transferred to new collection tube and the cup was washed thrice with lysis buffer and then transferred again to a fresh collection tube. 50 μl of 2X reducing sample buffer was added to resin and vortexed. The samples were heated for 5–10 min at 95–100 °C and then centrifuged at 6000 g for 2 min. Samples were then electrophoresed and immunoblotted.

### Silencing using siRNA

siRNA studies were performed on P338D1, a macrophage cell line. 0.5 Million cells were plated per well of 6 well tissue culture plates one day prior to transfection. Transfection was carried out according to manufacturer’s protocol (Santa Cruz biotechnology). Cells were transfected in serum-free media and after 6 h of transfection, 1 ml of complete media (RPMI 1640 supplemented with 10% FCS) was added. 12–14 h post transfection; media was replaced with complete media (RPMI 1640 supplemented with 10% FCS). After 48–60 h of transfection, cells were stimulated with anti-CD40. Control siRNA, H-Ras siRNA, N-Ras siRNA, K-Ras siRNA, Ras-GRP siRNA, Ras-GRF siRNA, Sos1/2 siRNA, Vav siRNA, Lyn siRNA, Syk siRNA transfection medium and transfection reagents were purchased from Santa Cruz biotechnology.

### Plasma membrane fractionation for assessing the activation of Sos-1/2 and Ras-GRP

6 million macrophages were stimulated with anti-CD40, washed twice in phosphate buffered saline after stimulation and resuspended in TES buffer (10 mM Tris-HCl (pH- 7.5), 1 mM EDTA, 0.25 M sucrose) with protease inhibitors and lysed by 20 passages of cell suspension through a 26-gauge needle. Nuclei were eliminated by centrifugation (20,000 X g; 15 mins) at 4 °C. The Supernatant was centrifuged (100,000 X g; 1 h) and obtained plasma membrane pellet was resuspended in TES buffer supplemented with 1% Nonidet P-40 and sonicated. Immunoblot analysis for translocated Sos or Ras-GRP was performed with the resuspended plasma membrane fractions [17].

### Sandwich ELISA

The culture supernatant obtained from P388D1 cells stimulated for 48 h and assayed for IL-12 and IL-10 by ELISA. Briefly, ELISA plates were coated overnight at 4 °C with purified anti–IL-10 (2 μg/ml) and IL-12 (2 μg/ml). Plates were washed three times (0.05% Tween 20 in PBS) and blocked for 2 h with blocking buffer (1% BSA). After washing the plates for three times, the plates were incubated overnight with standards or culture supernatants. Following this, plates were washed and incubated with respective biotin-conjugated detection antibodies for 1 h at 25 °C. Washed the plates again and incubated with peroxidase-conjugated streptavidin (Roche Applied Science) for 30 min followed by washing and color development using TMB substrate (BD Pharmingen, San Diego, CA). Reaction was stopped by the addition of 1 N H_2_SO_4_, and absorbance was measured at 450 nm. The values of standards were plotted and the quantity of the cytokines were estimated in the samples and expressed in pg/ml.

### Statistical analyses

All experiments were performed three times and the data corresponding to one representative experiment is shown here. Here the data are presented as mean ± SEM. Student’s t-test was used to compare the groups.

## Results

### Ras isoforms are differentially activated in CD40 signaling

**Fig. 1:**
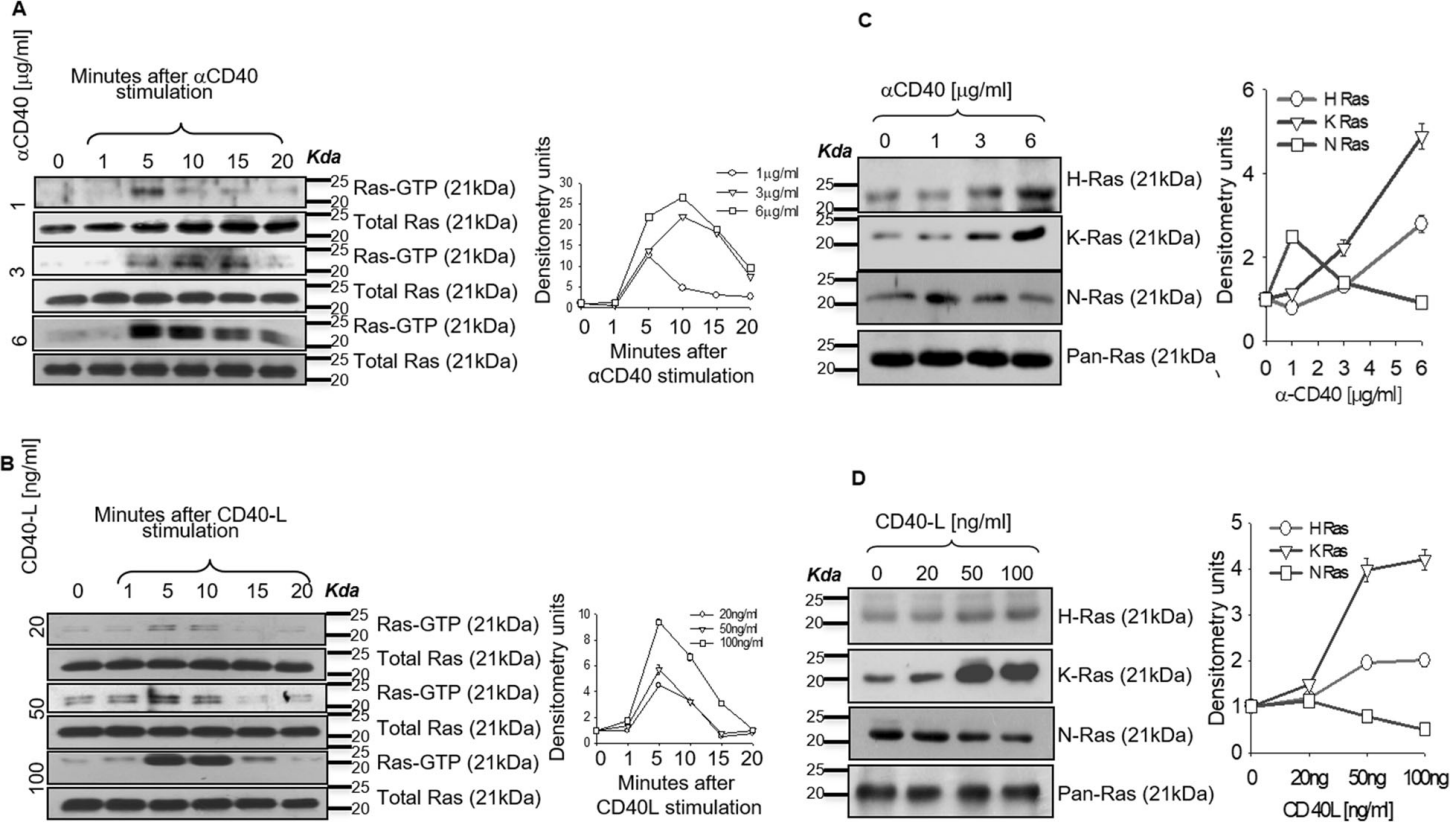
Ras isoforms are differentially activated in CD40 signaling. **a**, **b** Immunoblot analysis of activated and total Ras in thioglycolate-elicited BALB/c-derived macrophages stimulated with various concentrations of anti-CD40 for 1, 5, 10, 15 and 20 mins (**a**). **b**. Immunoblot analysis of activated and total Ras in thioglycolate-elicited BALB/c-derived macrophages stimulated with various concentrations of CD40 ligand for 1, 5, 10, 15 and 20 mins (**b**). Densitometric quantifications of the blots are shown (**c**, **d**) Immunoblot analysis of activated H, K and N-Ras and total Ras in macrophages untreated or stimulated with 1, 3, 6 μg/ml of anti-CD40 (**c**) or 20, 50, 100 ng of CD40 ligand (**d**) for 7 min. Densitometric quantifications of the blots are shown.

Previously we have shown that macrophage expressed CD40, induces activation of extracellular signal–regulated kinase-1/2 (ERK-1/2)-mediated anti-inflammatory IL-10 production and p38 mitogen-activated protein kinase (p38MAPK)-mediated pro-inflammatory IL-12 production, depending on the strength of signaling [11] reflecting a functional duality for CD40. Ras GTPases have been implied in CD40 signaling in B cells, endothelial cells, thymocytes and T-cells [13, 14, 18, 19]. In order to decipher signaling specificity of Ras isoforms in a receptor-driven system, we utilized reciprocal CD40 signaling in macrophages as a model and examined the activation of Ras isoforms. For this purpose, we performed the time kinetics of activation of Ras GTPase in BALB/c-derived thioglycolate-elicited peritoneal macrophages treated with different doses of anti-CD40 antibody (Fig. 1a) or recombinant CD40-ligand (Fig. 1b) as mentioned in the methods. The active form of Ras GTPases shows high affinity towards Ras-Binding Domain (RBD) of Raf kinases [20], therefore we immunoprecipitated the lysates with RBD peptides and probed for the activation of Ras. The maximal activation for Ras was obtained between 5 and 10 min. Further we treated peritoneal macrophages with different doses of stimulating anti-CD40 antibody or CD40 ligand for 7 min and pulled-down active Ras using RBD peptides and probed with Ras isoform-specific antibody. We observed that low strength of signaling with CD40 stimulating antibody and CD40 ligand activated primarily N-Ras whereas high strength of signaling activated H- and K-Ras (Fig. 1c and d). This observation indicated that Ras isoforms are differentially activated in CD40 stimulation.

### Ras isoforms differentially modulate reciprocal CD40 signaling

**Fig. 2:**
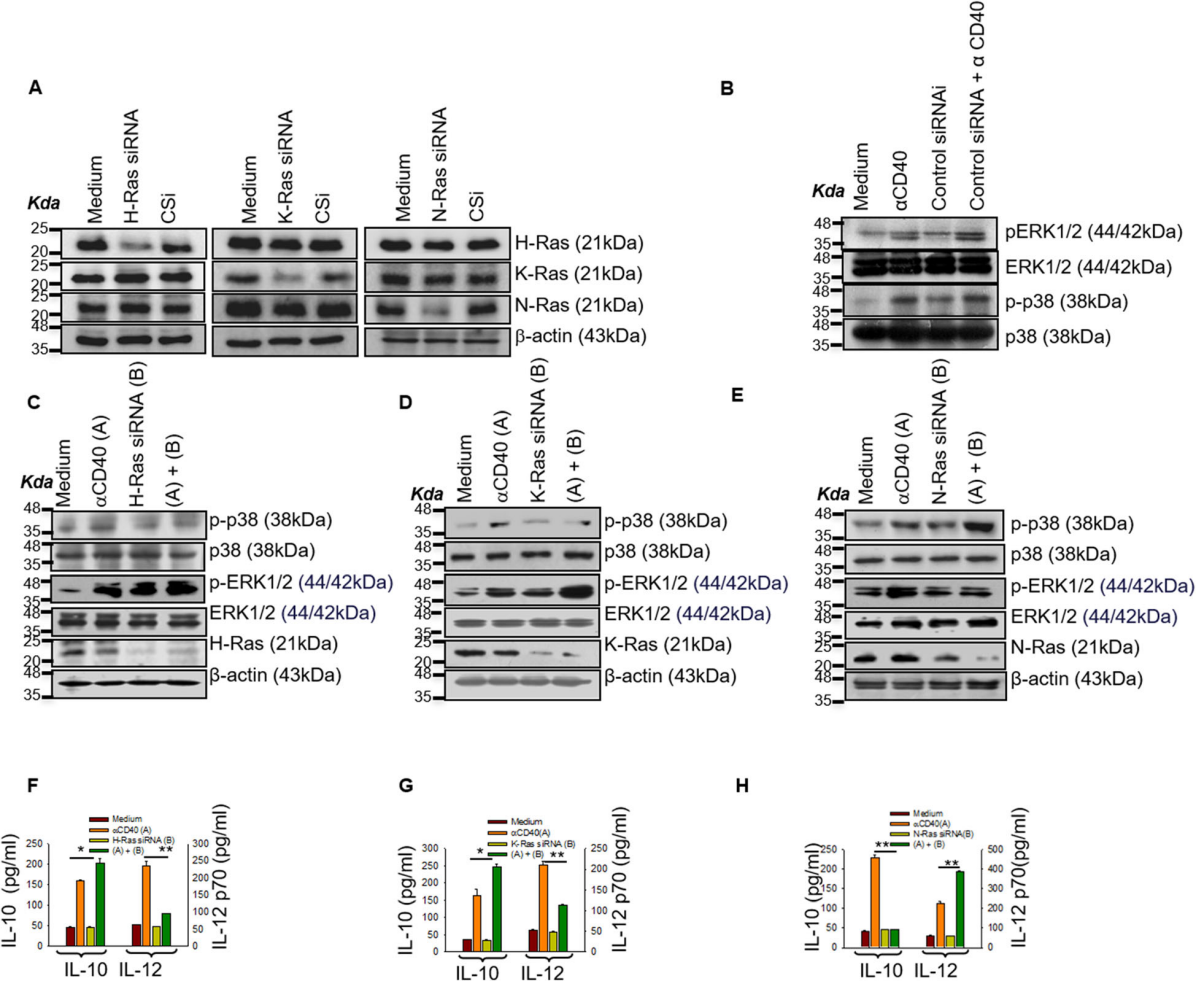
Ras isoforms differentially modulate reciprocal CD40 signaling. **a** Immunoblot analysis for checking the specificity of siRNA used for silencing respective isoforms. Only the Isoform-specific siRNA but not the control siRNA silenced the Ras isoforms. **b** Control siRNA treated P388D1 cells were stimulated with anti-CD40 antibody (3 μg/ml) for 15 min**;** using Western blot CD40 (3 μg/ml)-induced p38MAPK or ERK-1/2 activation was assessed. **c**-**e** siRNA treated P388D1 cells were stimulated with anti-CD40 antibody (3 μg/ml) for 15 min. siRNAs specific for H-Ras (**c**), K-Ras (**d**) and N-Ras (**e**) modulated the CD40 (3 μg/ml)-induced p38MAPK or ERK-1/2 activation, as assessed by Western blot. **e**-**g** siRNAs for H-Ras (**f**), K-Ras (**g**) and N-Ras (**h**) modulated the anti-CD40 antibody (3 μg/ml)-induced IL-12 or IL-10 productions as measured by using specific cytokine ELISA kits, in a macrophage-like mouse cell line P388D1. siRNA treated P388D1 cells were stimulated with anti-CD40 antibody (3 μg/ml) for 48 h. Data represented as mean ± SEM; *, *p* ≤ 0.05; ** *p* ≤ 0.005 compared with anti-CD40 stimulated P388D1 cells.

Although indicative of Ras GTPases involvement in CD40 signaling, these observations did not imply any differential roles for Ras isoforms in the CD40 signaling reciprocity [13, 14, 18, 19]. Therefore, we examined whether Ras isoform-specific small interfering RNA (siRNA) that significantly reduced the endogenous Ras isoform expression in P388D1, a macrophage-like cell line, would affect Anti-CD40 induced counteractive signaling. The inhibition was Ras isoform specific, but not control siRNA, as revealed by the criss-cross experiment (Fig. 2a and Additional file 1: Figure S1A). We also checked the effect of control siRNA on Anti-CD40 induced ERK1/2 and p38 MAPK activation. Control siRNA treatment did not alter the activation of ERK1/2 and p38 MAPK (Fig. 2b). H-Ras or K-Ras silencing using siRNA specific for Ras isoforms reduced CD40-induced p38MAPK activation but augmented ERK-1/2 activation (Fig. 2c and d; Additional file 1: Figure S1B and C). N-Ras silencing reduced CD40-induced ERK-1/2 activation but enhanced p38MAPK activation (Fig. 2e and Additional file 1: Figure S1D) indicating that the Ras isoforms are differentially associated with the CD40-induced reciprocal activation of p38MAPK or ERK-1/2.

As CD40 reciprocally signals through p38MAPK and ERK-1/2 to modulate the counteractive effectors IL-12 and IL-10, respectively, we examined whether suppression of the Ras isoforms’ expressions would modulate the production of these cytokines from P388D1 cells accordingly. We observed that silencing of H-Ras or K-Ras enhanced anti-CD40 induced IL-10 production but reduced IL-12 production (Fig. 2f and g). Whereas silencing N-Ras reduced anti-CD40 induced IL-10 production but reciprocally increased IL-12 production (Fig. 2h).

Taken together, these observations indicated that CD40 signaling through N-Ras activated ERK-1/2-mediated IL-10 production whereas the same receptor signaled through H-Ras and K-Ras to enhance p38MAPK-mediated IL-12 production. As IL-10 is an anti-inflammatory, immunosuppressive cytokine whereas IL-12 is a pro-inflammatory cytokine, these observations suggest that the modulation of Ras isoforms can be utilized for treatment strategies for inflammatory or immunosuppressive diseases.

### Ras isoforms differentially associate with Ras-guanine nucleotide exchange factor (Ras-GEF) in CD40 signaling

**Fig. 3:**
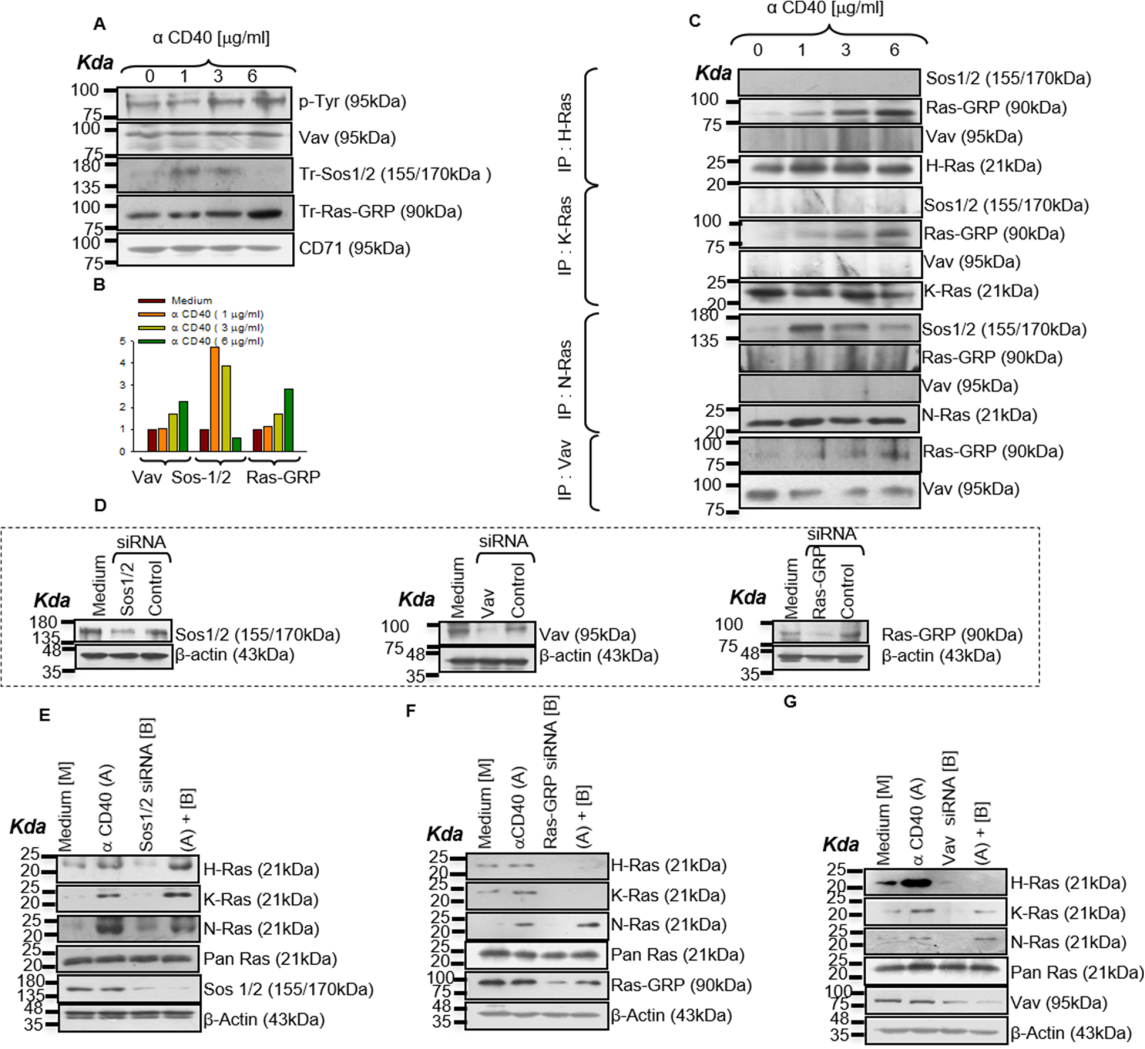
Ras isoforms differentially associate with Ras-guanine nucleotide exchange factor (Ras-GEF) in CD40 signaling. **a**, **b** CD40 activates the GEFs in a dose-dependent manner. Higher dose activates Ras-GRP and Vav whereas lower dose activates Sos-1/2. Immunoblot analysis of Vav or phospho tyrosine, CD71, translocated (Tr) Sos-1/2 or RasGRP in the lysates of macrophages treated with the indicated concentrations of anti-CD40 antibody (**a**). Densitometric quantifications of the blots are shown in the bottom panel (**b**). **c** Analysis of the association of Sos-1/2, Ras-GRP and Vav after immunoprecipitation with H-Ras, K-Ras and N-Ras in the lysates of macrophages treated with the indicated doses of anti-CD40 antibody. H-Ras and K-Ras associate with Ras-GRP whereas N-Ras associates with Sos-1/2 in macrophages stimulated with the indicated doses of anti-CD40 antibody. Also shown is the association of Ras-GRP with Vav by co-immunoprecipitation in the above-mentioned macrophage lysates. Vav associated with Ras GRP. Cells were stimulated for 3 min. **d** The siRNA for GEFs (Sos-1/2, Vav, and Ras-GRP) silenced respective GEFs in siRNA treated-inset. **e**-**g** Immunoblot analysis of total Ras and activated Ras isoforms in the lysates of un-transfected or Sos-1/2-, Vav-, Ras-GRP-specific siRNA transfected P388D1 cells stimulated with anti-CD40 (3 μg/ml) for 7 min. Sos-1/2 siRNA reduced N-Ras activation (**e**) whereas Vav siRNA and Ras-GRP siRNA reduced H-Ras and K-Ras activation (**f**, **g**).

As Ras isoforms’ activation is tightly regulated by the Ras-GEFs [21] we examined whether the Ras-GEFs’ (Sos-1/2, Vav, Ras-GRP, and Ras-GRF) [22–24] exhibited the same dose-dependent activation in response to CD40 stimulation. Ras-GRF was not observed to involve in CD40 signaling in macrophages (data not shown). We observed that higher doses of anti-CD40 antibody activated Ras-GRP and Vav whereas lower doses activated Sos-1/2 (Fig. 3a and b) suggesting differential associations between Ras-GEFs and Ras isoforms. Indeed, N-Ras co-immunoprecipitated with Sos-1/2, whereas Ras-GRP associated with H-Ras and K-Ras as a function of CD40 doses (Fig.3c). Ras isoforms were not detectably associated with Vav. As Vav-mediated Ras-GRP signaling is evolutionarily conserved and is important for Ras signaling [25], we tested the CD40-induced Vav-Ras-GRP association. We observed increased Vav-Ras-GRP association with increased doses of CD40 stimulation (Fig.3c; Lower Panel). Finally, transfection of P388D1 cells with Ras-GEF-specific siRNAs, which reduced the expression of respective endogenous Ras-GEFs (Fig. 3d insets), showed that Sos-1/2 (Fig. 3e and Additional file 1: Figure S2A) silencing diminished CD40-induced N-Ras activation whereas Ras-GRP (Fig. 3f and Additional file 1: Figure S2A) or Vav (Fig. 3g and Additional file 1: Figure S2A) silencing reduced CD40-induced H−/K-Ras activation suggesting that Sos-1/2 and Ras-GRP were providing signaling inputs to specific Ras isoforms. These results delineate the Ras isoforms signaling specificity through distinct complexes of Ras-GEFs and Ras isoforms leading to either p38MAPK or ERK-1/2 activation that are associated with the productions of pro-inflammatory cytokine IL-12 or anti-inflammatory cytokine IL-10, respectively.

### Differential activation of GEFs modulates CD40-induced counteractive effector functions

**Fig. 4:**
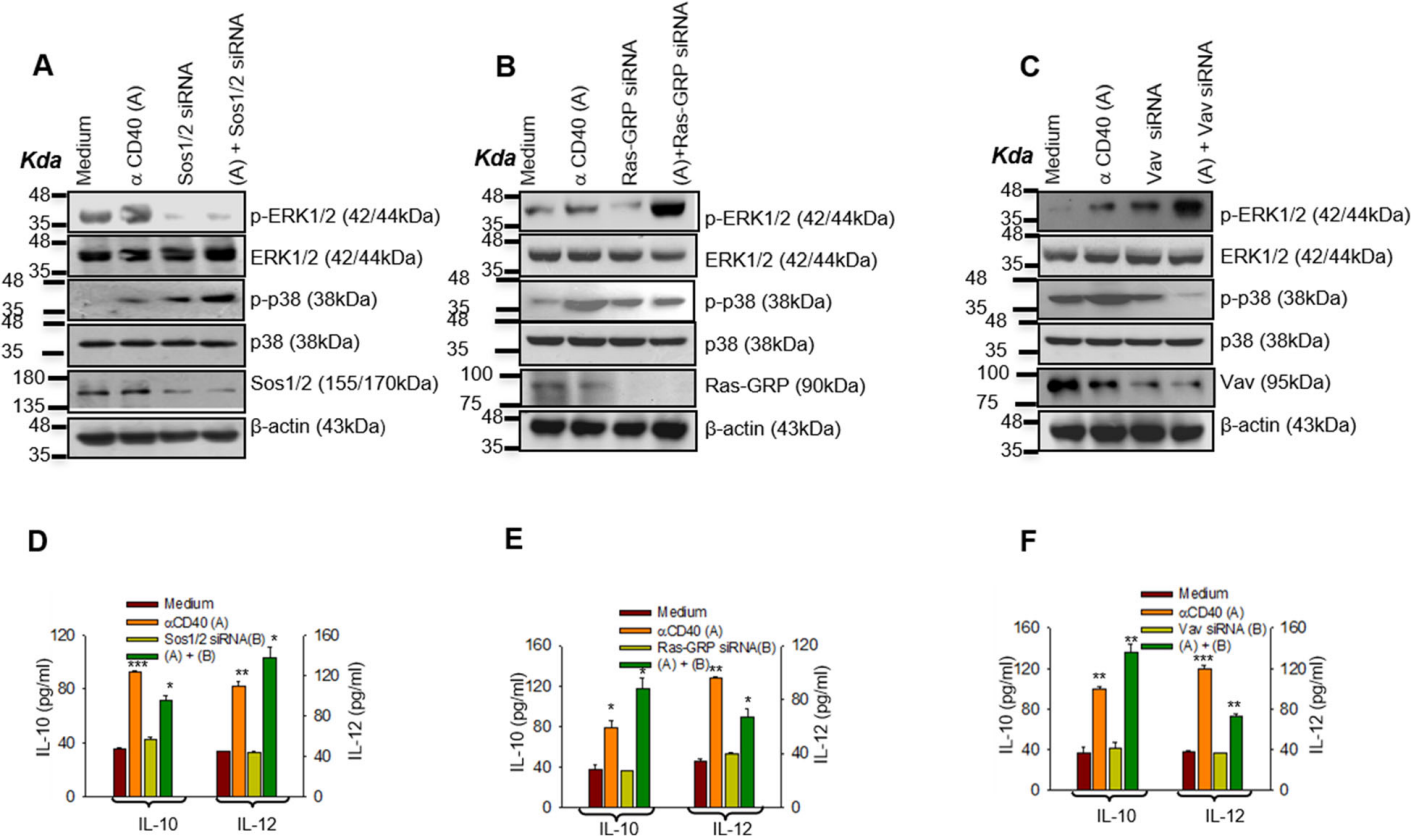
Differential activation of GEFs modulates CD40-induced counteractive effector functions. **a**-**c** Immunoblot analyses of total and phosphorylated (p-) p38MAPK and ERK-1/2 in the anti-CD40 antibody (3 μg/ml) stimulated P388D1 cells: un-transfected or transfected with Sos-1/2, Vav and Ras-GRP specific siRNA. siRNA treated P388D1 cells were stimulated with anti-CD40 antibody (3 μg/ml) for 15 min. Suppression of Sos-1/2 (**a**) reduced CD40 (3 μg/ml)-induced ERK-1/2 phosphorylation whereas silencing of Ras-GRP (**b**) and Vav (**c**) using respective siRNA reduced CD40 (3 μg/ml)-induced p38MAPK phosphorylation. **d**-**f** ELISA of IL-10 and IL-12p70 from the culture supernatants of the P388D1 cells transfected with the indicated GEF siRNA, followed by treatment with anti-CD40 antibody (3 μg/ml) for 48 h. respectively**.** *p* ≤ 0.05; ** *p* ≤ 0.005; *** *p* ≤ 0.0005 compared with control P388D1 cells.

As Ras-GEFs reciprocally regulate the CD40-induced activation of Ras isoforms, which were shown to reciprocally regulate counteractive effector functions such as IL-10 and IL-12 productions, we tested whether Ras-GEFs regulated the CD40-induced reciprocal regulation of these two cytokines. Sos-1/2 silencing reduced ERK-1/2 phosphorylation and IL-10 production but increased p38MAPK phosphorylation and IL-12 production (Fig. 4a and d; Additional file 1: Figure S2B). Whereas Ras-GRP/Vav silencing resulted in reverse effects on the CD40-induced MAPK phosphorylations and cytokine productions (Fig. 4b, c, e, f; Additional file 1: Figure S2B) in GEF-specific siRNAs-transfected P388D1 cells. While these results suggested reciprocal regulation of the counteractive effector functions – IL-10 and IL-12 productions- by Sos-1/2 and Vav/Ras-GRP, whether the Ras isoforms physically associate with CD40 differentially or how the Ras-GEFs are activated, as a function of the receptor stimulation remains unknown.

### Kinases Lyn and Syk selectively regulate Ras isoform’s activation through Ras-GEFs

**Fig. 5:**
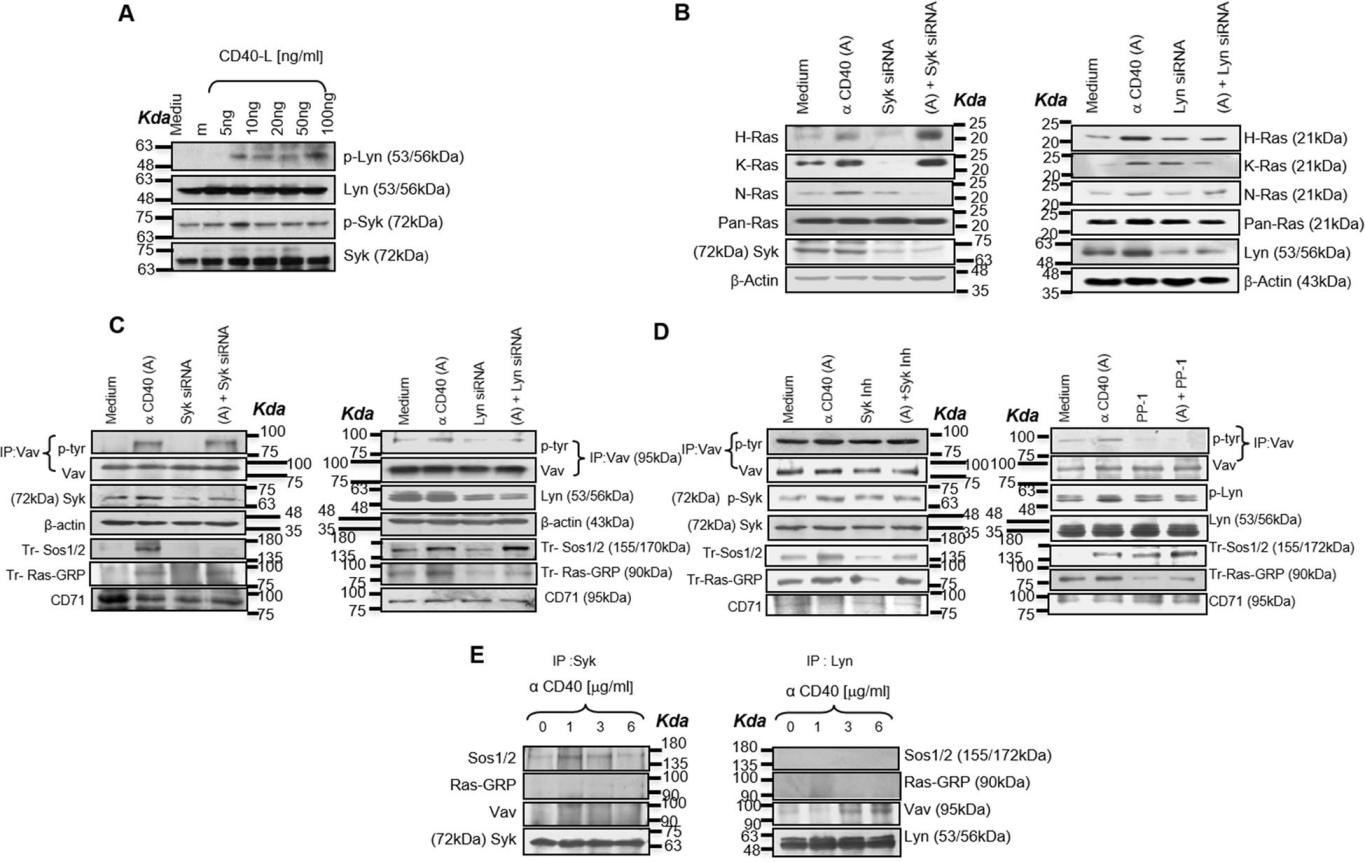
Kinases Lyn and Syk selectively regulate Ras isoform’s activation through Ras-GEFs. **a** CD40 activates the upstream kinases Lyn and Syk in a dose-dependent manner, with lower doses activating Syk and higher doses activating Lyn. **b** Immunoblot analysis of total Ras and activated Ras isoforms in the lysates of un-transfected or Syk- or Lyn-specific siRNA transfected, anti-CD40 antibody (3 μg/ml) treated P388D1 cells. **c** Immunoblot analyses of phospho-tyrosine (p-tyr-Vav)), and Vav translocated Sos-1/2 (Tr-Sos-1/2), translocated Ras-GRP (Tr-RasGRP), CD71, syk, lyn and β-actin in the lysates of untreated or Syk siRNA or Lyn siRNA or anti-CD40 antibody (3 μg/ml) treated P388D1 cells. **d** Immunoblot analysis of phospho-tyrosine (p-tyr Vav) and Vav translocated Sos-1/2, translocated Ras GRP, CD71, Lyn and phospho-Lyn, Syk and phospho-Syk (p-syk) in the lysates of untreated or Syk inhibitor (Syk Inh, 3 μM; Calbiochem, San Diego, CA) or PP-1 (340 nM; BIOMOL International, PA) treated and/or anti-CD40 antibody (3 μg/ml) treated macrophages. **e** Analysis of the association of Syk or Lyn with Sos-1/2, Vav, and Ras-GRP in the co-immunoprecipitates from the lysates of anti-CD40 antibody (1 μg/ml, 3 μg/ml and 6 μg/ml) treated macrophages.

Next, because CD40 activated Lyn and Syk (Fig. 5a, Additional file 1: Figure S3A), which is in line with their previously implicated roles in CD40-induced Ras-GTPase activation [13, 12], we examined whether Lyn and Syk selectively activated the GEFs. We observed that Syk silencing reduced CD40-induced N-Ras activation whereas Lyn silencing diminished H−/K-Ras activation (Fig. 5b; Additional file 1: Figure S3B). Because CD40-mediated Lyn or Syk activation is CD40-ligand dose-dependent and so are Ras-GEFs and Ras activation, we assessed whether Lyn or Syk silencing or the use of their inhibitors would differentially affect Ras-GEFs’ activation. Syk silencing in P388D1 cells and Syk inhibition [26] in macrophages reduced the CD40-induced Sos-1/2 activation, Lyn silencing by siRNA or inhibition by PP-1 [27] attenuated Vav or Ras-GRP activation (Fig. 5c, Fig. 5d; Additional file 1: Figure S3C and D). While Syk co-immunoprecipitated with Sos-1/2 at a low dose of anti-CD40 antibody (Fig. 5e). At high dose of anti-CD40, Lyn associated with Vav, but no association between Ras-GRP and Lyn was observed (Fig. 5e), suggesting that Lyn activates Vav which in turn associates with Ras-GRP to activate H−/K-Ras in CD40 signaling. Together, these data indicated CD40-induced differential association of Ras-GEFs with Lyn or Syk.

We further checked whether Lyn, Syk or CD40 has any direct association with Ras isoforms. Co-immunoprecipitation studies failed to detect Ras isoforms’ association with CD40 or Lyn or Syk (Additional file 1: Figure S3E). It seems plausible that following respective Ras-GEFs activation by Lyn or Syk in a complex, the activated Ras-GEFs dissociate and bind to specific Ras isoforms to continue the receptor signaling.

### Ras isoforms differ in their effector specificity and exhibit a difference in symmetry in functional site surface roughness

**Fig. 6:**
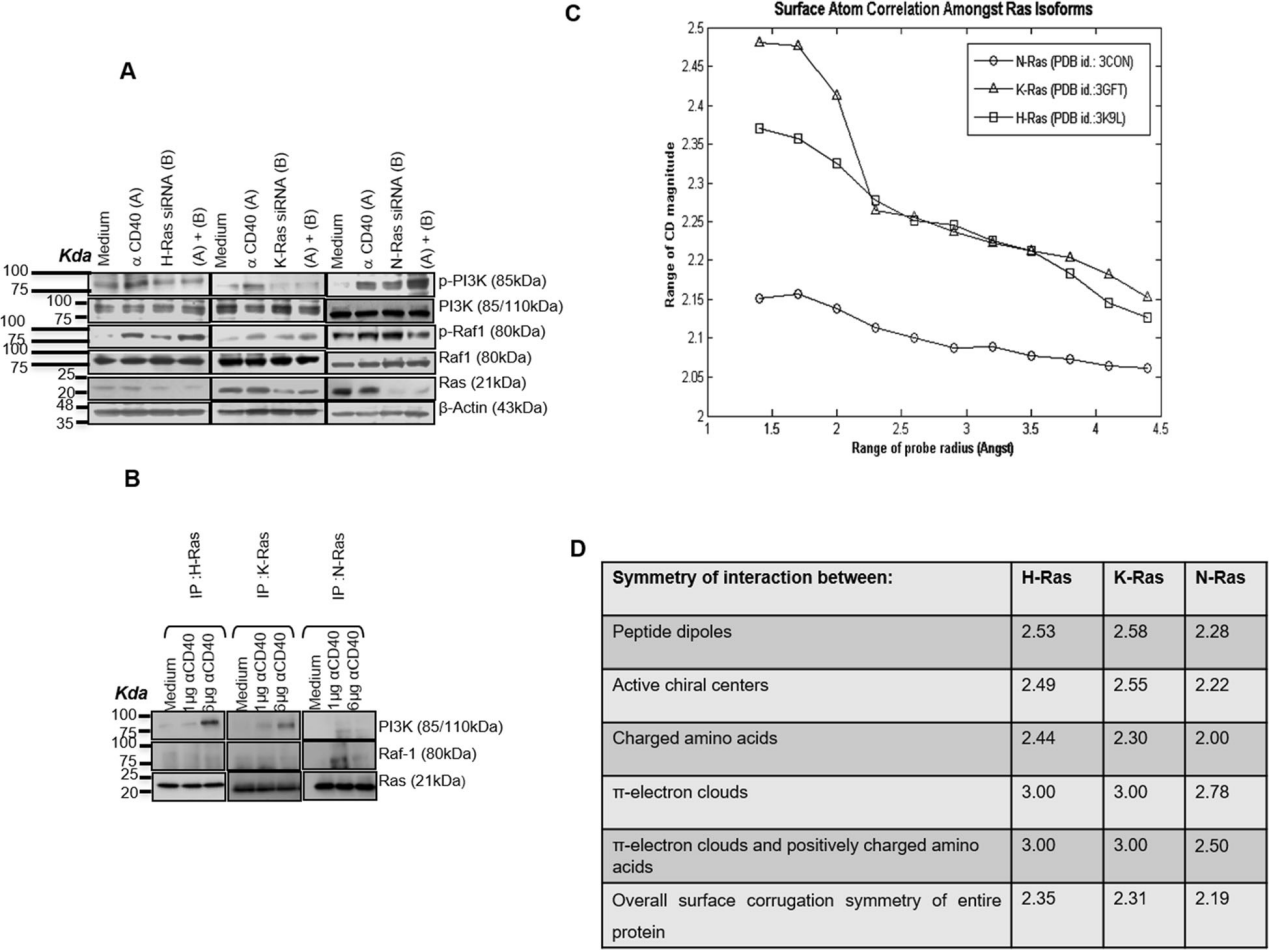
Ras isoforms differ in their effector specificity and Ras isoforms exhibit a difference in symmetry in functional site surface roughness. **a** H-Ras, K-Ras and N-Ras specific siRNAs modulated the anti-CD40 antibody (3 μg/ml)-induced downstream Ras effector proteins Raf-1 and PI-3 K in P388D1 cells, as assessed by Western blot. siRNA treated P388D1 cells were stimulated with anti-CD40 antibody (3 μg/ml) for 10 min. **b** Analysis of association of H-Ras, K-Ras or N-Ras with PI3K or Raf in the co-immunoprecipitates from the lysates of anti-CD40 antibody (1 μg/ml and 6 μg/ml) treated macrophages for 10 mins. **c** The Correlation Dimensions (CD) were calculated to quantify the dependency in the spatial organization of surface atoms. CD magnitude varies within 2.00 and 3.00 for protein space. Results of this analysis too point unambiguously to similar organizational principles between dependencies (correlations) amongst surface atoms of H-Ras and K-Ras and their marked difference to that in N-Ras isoform. **d** Investigations were carried out on representative structures, viz., on pdb id.: 3K9L for H-Ras, pdb id.: 3GFT for K-Ras and pdb id.: 3CON for N-Ras. Extent of symmetry for all six aforementioned biophysical properties show that H-Ras and K-Ras molecules have a similar scheme of structural organization in interior and exterior of the molecule, which differs from that observed in N-Ras molecule. Such differences are significant because sequence identity between H-Ras, K-Ras and N-Ras molecules is found to be ≈ 89%.

Because Raf-1 and PI-3 K are the downstream Ras-GTPase effectors with RBD, we next checked the effect of silencing of Ras isoforms on CD40-induced Raf-1 and PI-3 K phosphorylation. Ras isoforms were found to exhibit effector specificities as H- or K-Ras silencing lessened the receptor-induced PI-3 K activation and concomitantly increased Raf-1 activation while N-Ras silencing reduced Raf-1, but enhanced PI-3 K, activation (Fig. 6a; Additional file 1: Figure S4). H-Ras and K-Ras co-immunoprecipitated with PI3K upon stronger receptor stimulation, whereas N-Ras and Raf-1 co-immunoprecipitated with weaker receptor stimulation (Fig. 6b) indicating that Ras isoforms exhibit substrate specificity. Altogether, these observations demonstrate that Ras isoforms exhibit activator and effector specificities in a receptor-triggered signaling and effector functions.

The local and global patterns of surface roughness determine protein’s first level of interaction with its surroundings in terms of macromolecular recognition during protein-protein interactions [28] and any difference in the magnitude of surface differences can be mapped to functional differences in proteins [29]. The conventional three-dimensional structures of these Ras isoforms are not different (Additional file 1: Text 1 and Additional file 1: Table S1) due to homologous N-terminal residues (1–165 amino acids). Any subtle but significant differences in structural characteristics such as symmetry of interaction between peptide dipoles, active chiral centers, charged amino acids and π-electron clouds and also the interaction between π-electron clouds and positively charged amino acids cannot be technically probed. Surface roughness measured as fractal dimensions is a local, dynamic and contact-dependent property that can play a crucial role in determining protein-protein interactions [30]. Therefore, we compared the extent of symmetry of surface roughness in binding sites, in the atomic organization of entire protein exterior and in the distribution of the above-said biophysical characteristics in these isoforms by fractal dimension (FD)-based analyses [31]. We observed that the symmetry in functional site surface roughness for H-Ras (Surface_FD_ = 2.39) and K-Ras (Surface_FD_ = 2.39) are similar whereas that of N-Ras (Surface_FD_ = 2.25) is significantly different. The global profile of the surface atom organization, as quantified by correlation dimension, reiterated the inherent difference between H−/K-Ras and N-Ras surface atom organization (Fig. 6c, d). Similarly, various structural properties of H-Ras and K-Ras are comparable and both of them were significantly different from that of N-Ras (Additional file 1: Text 1, Additional file 1: Table S1, Additional file 1: Text 2, Additional file 1: Table S2,). These observations clearly hinted that Ras isoforms are distinct.

## Discussion

Here, we have delineated the molecular mechanism of differential activation of Ras isoforms induced by CD40. Low-dose anti-CD40 induced N-Ras activation requires syk-dependent Sos-1/2 activation, whereas high-dose anti-CD40 induced H-Ras or K-Ras activation requires lyn-dependent Vav- and Ras-GRP. Our observations establish for the first time that a single receptor can differentially activate Ras-GEFs that in turn, differentially regulate the three Ras isoforms, which are known to regulate diverse cellular processes in response to various extracellular stimuli. For instance, Ras is crucial for both positive and negative selection of T cells in thymus [32], as well as T cell proliferation and anergy [33, 34] but these studies considered Ras as one entity and did not distinguish between Ras isoforms. It is possible that Ras isoforms’ high sequence homology and identical effector functions in vitro had prompted two major generalizations- considering all Ras isoforms as a single Ras entity and H-Ras representing all Ras isoforms - that turned this concept of Ras isoform-specific functional duality far from obvious. The fractal studies show how the structural semblances between H-Ras and K-Ras isoforms conform to their functional similitude. By contrast, N-Ras isoform was structurally and functionally different from H−/K-Ras. Our findings that the fractal dimensions of H−/K-Ras and N-Ras are different and that Ras isoforms have activator and effector specificities raise the possibility of a novel drug development principle that eluded researchers for about four decades.

In order to dissect the role for individual Ras isoforms in counteractive effector functions, we needed a well worked out receptor system, which is known to use Ras as its signaling intermediate and also shown to result in such reciprocal functions. As CD40 satisfies both the criteria, we used CD40 as a model receptor to examine the mechanistic basis of distinctively opposite functions of Ras isoforms. Our findings revealed for the first time that one receptor differentially activated cellular Ras isoforms to modulate its reciprocal signaling outcome. With low doses of CD40 stimulation, N-Ras is preferentially activated, while with higher doses of CD40 stimulation, H- and K-Ras are activated. Also, our study showed that CD40 induced ERK-1/2 activation leading to anti-inflammatory IL-10 production is regulated by N-Ras, whereas p38MAPK activation leading to pro-inflammatory IL-12 production is H- or K-Ras dependent.

## Conclusion

In this study, we show that CD40 triggers differential activation of Ras isoforms to modulate its dual functioning. Our observations suggest that Ras isoforms’ activator and effector specificities, which are required for switching a receptor’s signaling from one module to its counteractive module, as a mechanism for the Ras-regulated functional duality of the receptor CD40.

## Supplementary information

**Additional file 1. Figure S1.** (A) Densitometry for immunoblot analysis for the silencing of Ras isoforms H, K, and N-Ras using specific siRNA. (B-D) Densitometry for immunoblots for phosphorylation of p38MAPK and ERK1/2 in P388D1 cells silenced for H (B), K (C), and N-Ras (D). **Figure S2**. (A) Densitometric analysis of the activation of H-Ras, K-Ras, and N-Ras on the silencing of Ras GEFs (Sos-1/2, Vav, and Ras-GRP) using GEF specific siRNA. (B) Densitometry of immunoblot analysis of phosphorylation of p38MAPK and ERK1/2 on silencing of Ras GEFs Sos-1/2, Vav and Ras-GRP. **Figure S3**. (A) Densitometric quantifications of the blots in Figure 5A. (B)Densitometric analyses of immunoblots of activated Ras isoforms in the lysates of untransfected or Syk or Lyn specific siRNA transfected, anti-CD40 antibody (3μg/ml) treated P388D1 cells, normalized to corresponding controls. (C) Densitometric analyses of immunoblots of translocated Sos-1/2 (Tr-Sos-1/2), translocated Ras-GRP (Tr-RasGRP), syk and lyn in the lysates of untreated or Syk siRNA or Lyn siRNA or anti-CD40 antibody (3μg/ml) treated P388D1 cells, normalized to corresponding controls. (D) Densitometric analyses of immunoblots of translocated Sos-1/2 (Tr-Sos-1/2), translocated Ras-GRP (Tr-Ras-GRP), phospho-lyn (p-lyn) and phospho-syk (p-syk) in the lysates of untreated or Syk inhibitor (Syk Inh, 3μM; Calbiochem, San Diego, CA) or PP-1 (340nM; BIOMOL International, PA) treated or anti-CD40 antibody (3μg/ml) treated macrophages, normalized to corresponding controls. (E) Co-immunoprecipitation of H-Ras, K-Ras and N-Ras at different doses of anti-CD40 to check for its association with Lyn, Syk and CD40. **Figure S4**. (A)Densitometry for effect of silencing of H-Ras, K-Ras, and N-Ras on the phosphorylation of PI3K and Raf. **Text 1.** Sequence and Structure Similarity Among Ras isoforms. **Table S1**. Sequence and structure similarity among Ras isoforms. **Text 2**. Comparative studies on symmetry of residue-residue interaction preferences, across Ras isoform structures. **Table S2**. Quantifying the symmetry in Residue-Residue interaction in three Ras isoforms.

## Data Availability

All data generated or analyzed during this study are included in this article [and its supplementary information files]. The materials described in our manuscript will be freely available for non-commercial purposes.

## Abbreviations

CD: Cluster of differentiation
CFC: Cardio-Facial Cutaneous
ERK-1/2: Extracellular Signal–Regulated Kinase-1/2
FBS: Fetal Bovine Serum
FD: Fractal Dimension
GDP: Guanosine Diphosphate
GEF: Guanine nucleotide Exchange Factor
GTP: Guanosine Triphosphate
H-Ras: Harvey Rat Sarcoma
HVR: Hyper variable Region
IL: Interleukin
K-Ras: Kirsten rat sarcoma
Lyn: Lck/Yes novel tyrosine kinase
N-Ras: Neuroblastoma Rat Sarcoma
p38MAPK: p38 mitogen-activated protein kinase
PI3K: Phosphatidyl inositol-3 kinase
Raf: Rapidly Accelerated Fibrosarcoma
Ras-GRF: Ras-Guanyl Releasing Factor
Ras-GRP: Ras-Guanyl Releasing Protein
RBD: Ras Binding Domain
SOS1/2: Son of Sevenless homolog 1/2
Syk: Spleen tyrosine kinase
