# Supplementary material for: Ras isoforms: signaling specificities in CD40 pathway
Source: Cell Commun Signal. 2020 Jan 6;18:3. doi: 10.1186/s12964-019-0497-1 (PMC6945409; doi:10.1186/s12964-019-0497-1)
Supplement: Supplementary file 1 — Additional file 1. Figure S1. (A) Densitometry for immunoblot analysis for the silencing of Ras isoforms H, K, and N-Ras using specific siRNA. (B-D) Densitometry for immunoblots for phosphorylation of p38MAPK and ERK1/2 in P388D1 cells silenced for H (B), K (C), and N-Ras (D). Figure S2. (A) Densitometric analysis of the activation of H-Ras, K-Ras, and N-Ras on the silencing of Ras GEFs (Sos-1/2, Vav, and Ras-GRP) using GEF specific siRNA. (B) Densitometry of immunoblot analysis of phosphorylation of p38MAPK and ERK1/2 on silencing of Ras GEFs Sos-1/2, Vav and Ras-GRP. Figure S3. (A) Densitometric quantifications of the blots in Figure 5A. (B)Densitometric analyses of immunoblots of activated Ras isoforms in the lysates of untransfected or Syk or Lyn specific siRNA transfected, anti-CD40 antibody (3μg/ml) treated P388D1 cells, normalized to corresponding controls. (C) Densitometric analyses of immunoblots of translocated Sos-1/2 (Tr-Sos-1/2), translocated Ras-GRP (Tr-RasGRP), syk and lyn in the lysates of untreated or Syk siRNA or Lyn siRNA or anti-CD40 antibody (3μg/ml) treated P388D1 cells, normalized to corresponding controls. (D) Densitometric analyses of immunoblots of translocated Sos-1/2 (Tr-Sos-1/2), translocated Ras-GRP (Tr-Ras-GRP), phospho-lyn (p-lyn) and phospho-syk (p-syk) in the lysates of untreated or Syk inhibitor (Syk Inh, 3μM; Calbiochem, San Diego, CA) or PP-1 (340nM; BIOMOL International, PA) treated or anti-CD40 antibody (3μg/ml) treated macrophages, normalized to corresponding controls. (E) Co-immunoprecipitation of H-Ras, K-Ras and N-Ras at different doses of anti-CD40 to check for its association with Lyn, Syk and CD40. Figure S4. (A)Densitometry for effect of silencing of H-Ras, K-Ras, and N-Ras on the phosphorylation of PI3K and Raf. Text 1. Sequence and Structure Similarity Among Ras isoforms. Table S1. Sequence and structure similarity among Ras isoforms. Text 2. Comparative studies on symmetry of residue-residue interaction preferences, [file 12964_2019_497_MOESM1_ESM.zip › 12964_2019_497_MOESM1_ESM/Additional file text and table.pdf]

Supplementary material

Supplementary figure legends

Supplementary Figure 1

(A) Densitometry for immunoblot analysis for the silencing of Ras isoforms H, K, and N-Ras using specific siRNA. **(B-D)** Densitometry for immunoblots for phosphorylation of p38MAPK and ERK1/2 in P388D1 cells silenced for H **(B)**, K **(C),** and N-Ras **(D).**

Supplementary Figure 2

(A) Densitometric analysis of the activation of H-Ras, K-Ras, and N-Ras on the silencing of Ras GEFs (Sos-1/2, Vav, and Ras-GRP) using GEF specific siRNA. **(B)** Densitometry of immunoblot analysis of phosphorylation of p38MAPK and ERK1/2 on silencing of Ras GEFs Sos-1/2, Vav and Ras-GRP.

Supplementary Figure 3

(A) Densitometric quantifications of the blots in Figure 5A. **(B)** Densitometric analyses of immunoblots of activated Ras isoforms in the lysates of untransfected or Syk or Lyn specific siRNA transfected, anti-CD40 antibody (3μg/ml) treated P388D1 cells, normalized to corresponding controls**. (C)** Densitometric analyses of immunoblots of translocated Sos-1/2 (Tr-Sos-1/2), translocated Ras-GRP (Tr-RasGRP), syk and lyn in the lysates of untreated or Syk siRNA or Lyn siRNA or anti-CD40 antibody (3μg/ml) treated P388D1 cells, normalized to corresponding controls. **(D)** Densitometric analyses of immunoblots of translocated Sos-1/2 (Tr-Sos-1/2), translocated Ras-GRP (Tr-Ras-GRP), phospho-lyn (p-lyn) and phospho-syk (p-syk) in the lysates of untreated or Syk inhibitor (Syk Inh, 3μM; Calbiochem, San Diego, CA) or PP-1 (340nM; BIOMOL International, PA) treated or anti-CD40 antibody (3μg/ml) treated macrophages, normalized to corresponding controls. **(E)** Co-immunoprecipitation of H-Ras, K-Ras and N-Ras at different doses of anti-CD40 to check for its association with Lyn, Syk and CD40.

Supplementary 4

Densitometry for effect of silencing of H-Ras, K-Ras, and N-Ras on the phosphorylation of PI3K and Raf.

Supplementary text 1

Sequence and Structure Similarity Among Ras isoforms

Though the sequences of H-Ras, K-Ras and N-Ras are similar to quite an extent, it was important to measure the exact extent of structural similarity among them. Amongst the representative structures (PDB-id.:3CON for N-Ras, PDB-id.:3GFT for K-Ras, and PDB-id.:3K9L for H-Ras), 3CON is single-chained, whereas 3GFT contains 6 chains and 3CON has 3 chains. To ensure a rigorous assessment of structural similarity, all 27 combinations of chain-specific flexible structural alignments were conducted. This is done using FATCAT (Flexible structure AlignmenT by Chaining Aligned fragment pairs allowing Twists) [S1-1]. The results are presented in Supplementary-Table-2.

Very low magnitude of RMSD (<1.0 Å) among the three molecules signified the astounding structural similarities among the three Ras isoforms. The sequence identity was found to be > 88% and the sequence similarity was found to be > 94%.

Supplementary Table-1

Sequence and structure similarity among Ras isoforms

PROTEIN-1	PROTEIN-1 LENGTH	PROTEIN-2	PROTEIN-2 LENGTH	P-VALUE	RAW-SCORE	RMSD	%Seq. IDENTITY	%Seq. SIMILARITY
H-Ras(PDB Id.: 3K9L(A))	3K9L(A)-Length:159	K-Ras(PDB Id.: 3GFT(A))	3GFT(A)-Length:167	0.00E+00	435.51	0.94	88.6	93.9
H-Ras(PDB Id.: 3K9L(A))	3K9L(A)-Length:159	K-Ras(PDB Id.: 3GFT(B))	3GFT(B)-Length:166	0.00E+00	424.81	0.75	88.9	94.4
H-Ras(PDB Id.: 3K9L(A))	3K9L(A)-Length:159	K-Ras(PDB Id.: 3GFT(C))	3GFT(C)-Length:165	0.00E+00	411.2	0.7	87.6	93.2
H-Ras(PDB Id.: 3K9L(A))	3K9L(A)-Length:159	K-Ras(PDB Id.: 3GFT(D))	3GFT(D)-Length:150	0.00E+00	375.56	0.61	87.4	92.5
H-Ras(PDB Id.: 3K9L(A))	3K9L(A)-Length:159	K-Ras(PDB Id.: 3GFT(E))	3GFT(E)-Length:163	0.00E+00	426.89	0.69	91.2	96.7
H-Ras(PDB Id.: 3K9L(A))	3K9L(A)-Length:159	K-Ras(PDB Id.: 3GFT(F))	3GFT(F)-Length:155	0.00E+00	374.96	0.64	87.4	92.4
H-Ras(PDB Id.: 3K9L(B))	3K9L(B)-Length:158	K-Ras(PDB Id.: 3GFT(A))	3GFT(A)-Length:167	0.00E+00	426.05	1.48	88.6	94
H-Ras(PDB Id.: 3K9L(B))	3K9L(B)-Length:158	K-Ras(PDB Id.: 3GFT(B))	3GFT(B)-Length:166	0.00E+00	430.52	1.48	90.7	96.3
H-Ras(PDB Id.: 3K9L(B))	3K9L(B)-Length:158	K-Ras(PDB Id.: 3GFT(C))	3GFT(C)-Length:165	0.00E+00	422.27	1.2	87.6	93.2
H-Ras(PDB Id.: 3K9L(B))	3K9L(B)-Length:158	K-Ras(PDB Id.: 3GFT(D))	3GFT(D)-Length:150	0.00E+00	375.45	0.64	87.3	92.4
H-Ras(PDB Id.: 3K9L(B))	3K9L(B)-Length:158	K-Ras(PDB Id.: 3GFT(E))	3GFT(E)-Length:163	0.00E+00	432.47	1.43	92.5	96.1
H-Ras(PDB Id.: 3K9L(B))	3K9L(B)-Length:158	K-Ras(PDB Id.: 3GFT(F))	3GFT(F)-Length:155	0.00E+00	399.86	0.78	89.2	94.3
H-Ras(PDB Id.: 3K9L(C))	3K9L(C)-Length:158	K-Ras(PDB Id.: 3GFT(A))	3GFT(A)-Length:167	0.00E+00	429.81	1.28	88.6	94
H-Ras(PDB Id.: 3K9L(C))	3K9L(C)-Length:158	K-Ras(PDB Id.: 3GFT(B))	3GFT(B)-Length:166	0.00E+00	433.8	1.22	90.7	96.3
H-Ras(PDB Id.: 3K9L(C))	3K9L(C)-Length:158	K-Ras(PDB Id.: 3GFT(C))	3GFT(C)-Length:165	0.00E+00	420.48	0.97	88.9	94.4
H-Ras(PDB Id.: 3K9L(C))	3K9L(C)-Length:158	K-Ras(PDB Id.: 3GFT(D))	3GFT(D)-Length:150	0.00E+00	380.69	0.59	88	93
H-Ras(PDB Id.: 3K9L(C))	3K9L(C)-Length:158	K-Ras(PDB Id.: 3GFT(E))	3GFT(E)-Length:163	0.00E+00	442.05	1.1	92.5	96.1
H-Ras(PDB Id.: 3K9L(C))	3K9L(C)-Length:158	K-Ras(PDB Id.: 3GFT(F))	3GFT(F)-Length:155	0.00E+00	400.91	0.61	89.2	94.3
N-Ras(PDB Id.: 3CON)	3CON-Length:156	K-Ras(PDB Id.: 3GFT(A))	3GFT(A)-Length:167	0.00E+00	410.44	0.89	85.6	91.6
N-Ras(PDB Id.: 3CON)	3CON-Length:156	K-Ras(PDB Id.: 3GFT(B))	3GFT(B)-Length:166	0.00E+00	412.3	0.93	87.7	93.9
N-Ras(PDB Id.: 3CON)	3CON-Length:156	K-Ras(PDB Id.: 3GFT(C))	3GFT(C)-Length:165	0.00E+00	406.3	0.91	86.5	92.6
N-Ras(PDB Id.: 3CON)	3CON-Length:156	K-Ras(PDB Id.: 3GFT(D))	3GFT(D)-Length:150	0.00E+00	402.16	0.77	87.8	94.2
N-Ras(PDB Id.: 3CON)	3CON-Length:156	K-Ras(PDB Id.: 3GFT(E))	3GFT(E)-Length:163	0.00E+00	412.92	0.91	89.4	95.6
N-Ras(PDB Id.: 3CON)	3CON-Length:156	K-Ras(PDB Id.: 3GFT(F))	3GFT(F)-Length:155	0.00E+00	374.37	0.72	86.1	92.4
N-Ras(PDB Id.: 3CON)	3CON-Length:156	H-Ras(PDB Id.: 3K9L(A))	3K9L(A)-Length:159	0.00E+00	424.71	0.68	88.1	94.3
N-Ras(PDB Id.: 3CON)	3CON-Length:156	H-Ras(PDB Id.: 3K9L(B))	3K9L(B)-Length:158	0.00E+00	400.01	0.64	88	94.3
N-Ras(PDB Id.: 3CON)	3CON-Length:156	H-Ras(PDB Id.: 3K9L(C))	3K9L(C)-Length:158	0.00E+00	409.94	1.25	88.6	94.9
					Mean = 410.9[illegible]	Mean = 0.92	Mean = 88.62	Mean = 94.34

Legend for Supplementary-Table-1

P-value: P-value denotes the probability that two structures are similar. According to FATCAT, a pair with <0.05, has significant structural similarity.

RMSD: It is the measure of average distance between the atoms of superimposed proteins. Magnitude of <1.00 RMSD implies exceptionally good structural similarity between two proteins.

Reference- Ye, Y. and Godzik, A., 2003. Flexible structure alignment by chaining aligned fragment pairs allowing twists. *Bioinformatics*, *19*(suppl_2), pp.ii246-ii255.

Supplementary text 2

Comparative studies on symmetry of residue-residue interaction preferences, across Ras isoform structures

Since the numerous differences in macroscopic biophysical properties are rooted in the subtle differences in spatial arrangement of amino acids in native state of protein structures, effort was directed to quantify the symmetry of self-similarity in pattern of residue-residue coupling, existing in three Ras isoform molecules. Though the structural similarity across the isoforms is extremely high (Supplementary-Table-1), such bottom-up investigation of residue interaction profile, employing the same tool-set (namely, correlation dimension (CD)) as used for macroscopic distributions, detected significant differences in residue preferences. For example, $CD_{Ala-Ala}$(N-Ras) = 2.63, but $CD_{Ala-Ala}$(K-Ras) = 2.36 and $CD_{Ala-Ala}$(H-Ras) = 2.35. Similarly one finds $CD_{Ala-Thr}$(N-Ras) = 2.03, but $CD_{Ala-Thr}$(K-Ras) = 2.30 and $CD_{Ala-Thr}$(H-Ras) = 2.31; $CD_{Ala-Gln}$(N-Ras) = 2.16, $CD_{Ala-Gln}$(K-Ras) = 2.45, $CD_{Ala-Gln}$(H-Ras) = 2.45; $CD_{Glu-Cys}$(N-Ras) = 2.58, $CD_{Glu-Cys}$(K-Ras) = 2.15, $CD_{Glu-Cys}$(H-Ras) = 2.12; etc. Details of these interactions can be found in Supplementary-Table-2.

Interestingly, the trend observed in symmetries of macroscopic distributions, viz. H-Ras and K-Ras molecules sharing similar profiling, as contrasted to the one characterizing N-Ras molecule – is observed in many of (microscopic) symmetries of residue preferences. Thus it probably won't be wrong to hypothesize that the differences in residue interaction profiles cumulate in a non-linear manner to gives rise to differences at the macroscopic profile of property distribution symmetries.

Supplementary Table-2

Quantifying the symmetry in Residue-Residue interaction in three Ras isoforms

H-RAS	PDB Id.: 3K9L																			
	ala	cys	met	thr	pro	gln	asn	ser	arg	his	lys	phe	tyr	trp	glu	asp	val	leu	gly	ile
ala	2.353616	NULL	3.299017	2.310152	3.075448	2.455825	1.906902	2.140314	2.60702	NULL	2.194987	1.986103	2.322277	0	3.286799	2.751773	2.310443	2.254489	2.016702	2.765809
cys	0	NULL	1.26186	2.207151	NULL	2.200864	2.423592	2.647632	2.982986	NULL	2.771244	2.049214	2.049214	0	2.120395	3.319023	1.813588	1.958618	2.523719	2.402174
met	0	0	NULL	1.958618	1.36907	2.022202	NULL	NULL	2.203114	NULL	2.680144	1.726833	2.59219	0	2.200864	4.227507	2.189528	1.935531	2.203114	2.299017
thr	0	0	0	2.669435	2.504555	2.750614	2.039582	2.375788	2.869145	NULL	2.316103	2.862802	2.707669	0	2.295243	2.710763	2.388738	2.744858	2.685451	2.474262
pro	0	0	0	0	NULL	3.12575	NULL	3.840761	3.48498	1.813588	NULL	1.929947	2.618199	0	1.8387	2.695975	2.686113	2.726833	2.664033	1.927142
gln	0	0	0	0	0	2.362303	2.53983	2.655869	2.009735	1.692043	2.714335	2.868771	2.603285	0	2.633587	2.831512	2.673019	2.541744	3.220478	2.372808
asn	0	0	0	0	0	0	1.464974	2.26186	2.343428	2.12575	2.054522	2.043875	4.043875	0	3.209832	3.942003	2.159377	2.584832	2.799691	2.095903
ser	0	0	0	0	0	0	0	NULL	2.320605	NULL	1.874644	2.659377	2.452755	0	2.482148	1.730961	2.581716	2.676143	2.330589	2.440371
arg	0	0	0	0	0	0	0	0	2.986414	2.311074	2.427816	4.619622	2.3591	0	2.155566	2.505435	2.760343	2.783205	3.065045	2.269951
his	0	0	0	0	0	0	0	0	0	NULL	NULL	2.680144	2.785579	0	2.464974	2.456263	2.905344	NULL	NULL	2.200864
lys	0	0	0	0	0	0	0	0	0	0	2.785579	3.006718	3.005601	0	2.726833	2.020688	2.203766	2.338212	1.953494	2.701991
phe	0	0	0	0	0	0	0	0	0	0	0	2.542487	2.993232	0	2.36907	2.441928	1.85613	1.784155	2.831793	2.347752
tyr	0	0	0	0	0	0	0	0	0	0	0	0	4.022202	0	2.634535	2.206479	2.709909	2.488272	2.774849	1.980836
trp	0	0	0	0	0	0	0	0	0	0	0	0	0	0	0	0	0	0	0	0
glu	0	0	0	0	0	0	0	0	0	0	0	0	0	0	4.043875	2.578902	2.619439	2.880058	2.53844	2.422836
asp	0	0	0	0	0	0	0	0	0	0	0	0	0	0	0	2.684489	2.783123	2.652912	3.458206	2.678059
val	0	0	0	0	0	0	0	0	0	0	0	0	0	0	0	0	2.249129	2.101853	2.202664	2.367463
leu	0	0	0	0	0	0	0	0	0	0	0	0	0	0	0	0	0	1.831793	2.802634	2.222105
gly	0	0	0	0	0	0	0	0	0	0	0	0	0	0	0	0	0	0	1.806034	2.670973
ile	0	0	0	0	0	0	0	0	0	0	0	0	0	0	0	0	0	0	0	3.218712

K-RAS	PDB Id.: 3GFT																			
	ala	cys	met	thr	pro	gln	asn	ser	arg	his	lys	phe	tyr	trp	glu	asp	val	leu	gly	ile
ala	2.357763	NULL	4.47836	2.301244	3.205359	2.447745	2.22312	2.240934	3.102878	NULL	2.260134	1.820458	2.617524	0	4.806666	2.569114	2.079303	2.396739	1.920799	2.866016
cys	0	NULL	1.348615	2.405589	NULL	2.635134	2.726833	2.778439	3.251689	NULL	2.871471	2.299017	2.268159	0	2.152615	3.557838	1.973118	2.026578	2.664033	2.322972
met	0	0	NULL	2.376817	2.33985	2.007196	NULL	4.033103	1.994031	NULL	2.701191	1.834044	2.219515	0	2.384267	3.214283	2.295701	2.129935	1.931529	2.344666
thr	0	0	0	2.750477	2.877491	2.588712	2.22312	2.392269	2.718366	3.270249	2.530161	2.897723	2.505247	0	2.230287	2.667344	2.580409	2.863137	2.734212	2.705015
pro	0	0	0	0	NULL	NULL	3.672124	2.929947	3.071301	2.246561	NULL	2.322972	3.209832	0	1.886446	2.771244	2.649698	2.334718	2.803185	2.133444
gln	0	0	0	0	0	2.490616	2.796183	3.061901	2.35923	1.944817	2.629184	2.528259	3.023045	0	3.115909	2.696897	3.080605	2.666013	3.842099	2.691254
asn	0	0	0	0	0	0	1.892789	2.111826	2.528876	2.412946	2.461397	2.351045	2.880058	0	3.204462	3.043051	2.251263	2.711535	2.779872	2.105961
ser	0	0	0	0	0	0	0	NULL	2.49482	NULL	2.238578	2.956853	2.771244	0	2.618953	2.099537	2.771244	2.53983	2.498727	2.372551
arg	0	0	0	0	0	0	0	0	2.828973	3.22312	2.454201	NULL	2.275305	0	2.289869	2.412122	2.813588	2.700245	3.115909	2.284508
his	0	0	0	0	0	0	0	0	0	3.405992	2.731373	2.947972	2.112304	0	1.942003	2.193873	3.146858	NULL	NULL	2.122809
lys	0	0	0	0	0	0	0	0	0	0	3.321282	3.695975	3.877919	0	2.265843	2.544504	2.551729	2.697181	2.247059	2.65996
phe	0	0	0	0	0	0	0	0	0	0	0	2.965647	3.252328	0	2.565417	2.707803	2.02342	1.87201	2.597124	2.217164
tyr	0	0	0	0	0	0	0	0	0	0	0	0	NULL	0	2.685763	2.311672	2.705786	2.431421	2.614529	2.103784
trp	0	0	0	0	0	0	0	0	0	0	0	0	0	0	0	0	0	0	0	0
glu	0	0	0	0	0	0	0	0	0	0	0	0	0	0	3.63093	2.776047	2.949161	2.86036	2.694892	2.379655
asp	0	0	0	0	0	0	0	0	0	0	0	0	0	0	0	3.372808	2.843254	2.999112	3.229692	3.094779
val	0	0	0	0	0	0	0	0	0	0	0	0	0	0	0	0	2.411755	2.149657	2.294701	2.357763
leu	0	0	0	0	0	0	0	0	0	0	0	0	0	0	0	0	0	1.923858	2.926908	2.217607
gly	0	0	0	0	0	0	0	0	0	0	0	0	0	0	0	0	0	0	1.815884	2.659377
ile	0	0	0	0	0	0	0	0	0	0	0	0	0	0	0	0	0	0	0	3.991533

N-RAS	PDB Id.: 3CON																			
	ala	cys	met	thr	pro	gln	asn	ser	arg	his	lys	phe	tyr	trp	glu	asp	val	leu	gly	ile
ala	2.63093	NULL	NULL	2.033103	2.551729	2.161732	1.9372	1.73814	3.464974	1.26186	1.784155	1.500674	2.242696	0	2.480019	2.56813	2	2.191807	1.892789	2.500674
cys	0	0	0	2.182658	NULL	2.182658	NULL	NULL	NULL	0	NULL	1.26186	1.63093	0	2.578902	NULL	1.228756	1.551729	2.464974	2.049214
met	0	0	0	1.813588	1.140314	1.834044	NULL	NULL	2.813588	0	2.680144	1.63093	2.63093	0	2.22312	NULL	1.974358	1.63093	1.929947	1.892789
thr	0	0	0	2.680144	2.182658	2.182658	2.182658	2.385772	2.320605	1.771244	2.095903	2.418284	2.664033	0	2.134101	2.354506	2.236217	2.515555	2.383405	2.32466
pro	0	0	0	0	0	NULL	NULL	NULL	2.813588	0	NULL	2.140314	1.892789	0	1.59219	2.49482	2.504555	2.311074	2.26186	2.065045
gln	0	0	0	0	0	2.095903	2.049214	3.033103	1.974358	NULL	2.334718	2.49482	2.551729	0	2.423592	2.578902	2.205999	1.985436	3.664033	2.113928
asn	0	0	0	0	0	0	1.892789	1.668088	1.680144	NULL	2.209832	2.033103	2.095903	0	3.12575	3.357763	2.065045	2.280628	2.48498	1.726833
ser	0	0	0	0	0	0	0	2.402174	3.311074	NULL	1.560877	2.311074	2.236217	0	2.834044	1.787355	2.283278	2.565417	2.291706	2.148942
arg	0	0	0	0	0	0	0	0	2.22312	NULL	2.578902	NULL	1.813588	0	1.464974	2.551729	2.73589	2.965647	3.741879	1.86389
his	0	0	0	0	0	0	0	0	0	0	2.459432	1.464974	NULL	0	3.321928	NULL	NULL	2	NULL	NULL
lys	0	0	0	0	0	0	0	0	0	0	2.402174	2.523719	3.542487	0	2.30627	2.061113	2.26186	2.373874	2.022202	2.427816
phe	0	0	0	0	0	0	0	0	0	0	0	2.464974	3.26186	0	2.444518	2.664033	1.698386	1.617033	2.464974	2.21517
tyr	0	0	0	0	0	0	0	0	0	0	0	0	NULL	0	1.929947	1.849089	2.32466	2.134101	3.154649	1.497486
trp	0	0	0	0	0	0	0	0	0	0	0	0	0	0	0	0	0	0	0	0
glu	0	0	0	0	0	0	0	0	0	0	0	0	0	0	2.402174	2.311074	2.326904	2.213172	2.357763	2.311074
asp	0	0	0	0	0	0	0	0	0	0	0	0	0	0	0	3.423592	2.624587	2.441928	3.173417	2.334718
val	0	0	0	0	0	0	0	0	0	0	0	0	0	0	0	0	2.145117	1.865287	2.046015	2.175736
leu	0	0	0	0	0	0	0	0	0	0	0	0	0	0	0	0	0	1.63093	2.719216	1.918675
gly	0	0	0	0	0	0	0	0	0	0	0	0	0	0	0	0	0	0	1.69373	2.689675
ile	0	0	0	0	0	0	0	0	0	0	0	0	0	0	0	0	0	0	0	2.726833

Legend for Supplementary-Table-2

Correlation Dimension (CD) calculations were done using Grassberger-Procaccia algorithm (S2-1). In some cases, the CD magnitude of particular residue-residue pair show <2.00 or >3.00 magnitude. These occur due to insufficient nature of statistical sample (viz., less than adequate number of residues), which leads to insufficient number of data points and finally, cause aberration. Such cases should not be taken into consideration, because, while CD=2.00 implies total absence of

symmetry in interactions (viz., the least space-filling case) and CD=3.00 implies maximum possible symmetric nature of interactions (viz., the maximum space-filling case); CD > 3.00 or CD < 2.00 for protein structure (a 3-D entity) do not hold much biological meaning.

Reference- Grassberger, P. and Procaccia, I., 1983. Measuring the strangeness of strange attractors. *Physica D: Nonlinear Phenomena*, *9*(1-2), pp.189-208.
