# Supplementary figures and images for: Ras isoforms: signaling specificities in CD40 pathway
Source: Cell Commun Signal. 2020 Jan 6;18:3. doi: 10.1186/s12964-019-0497-1 (PMC6945409; doi:10.1186/s12964-019-0497-1)

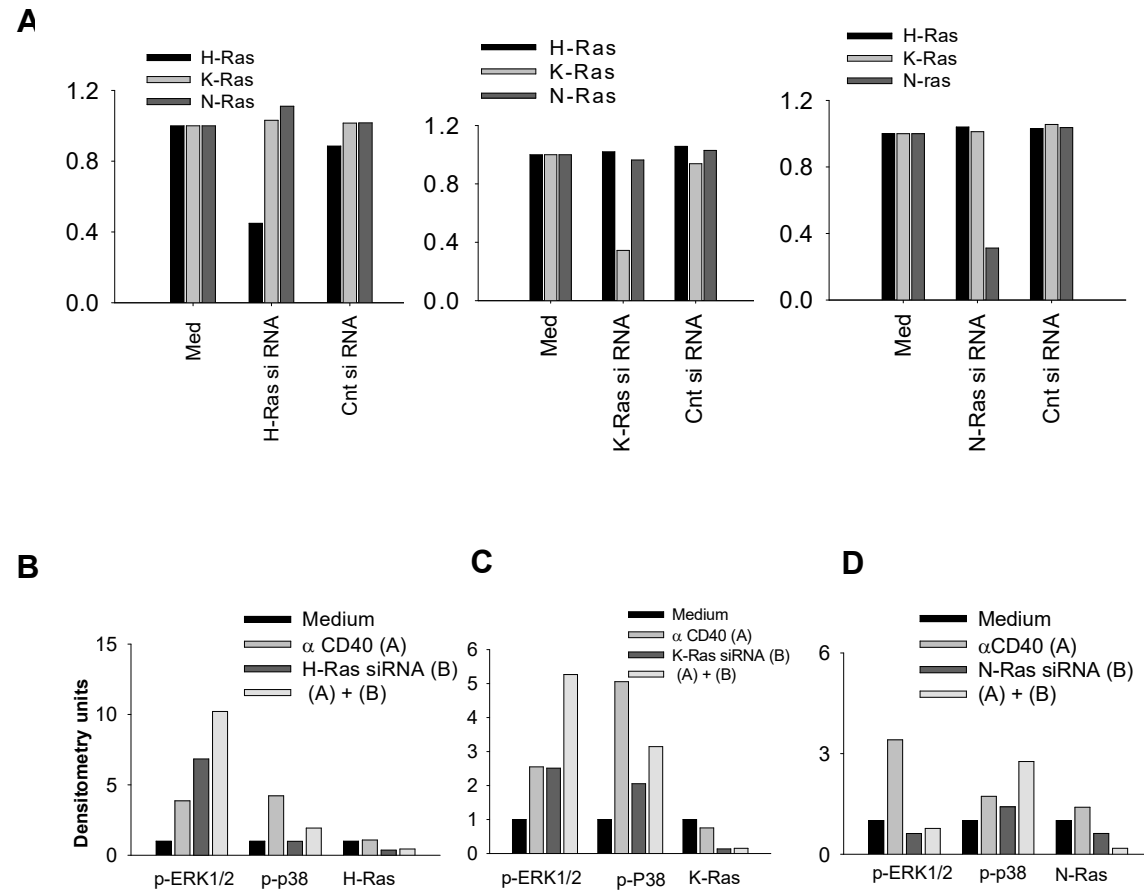

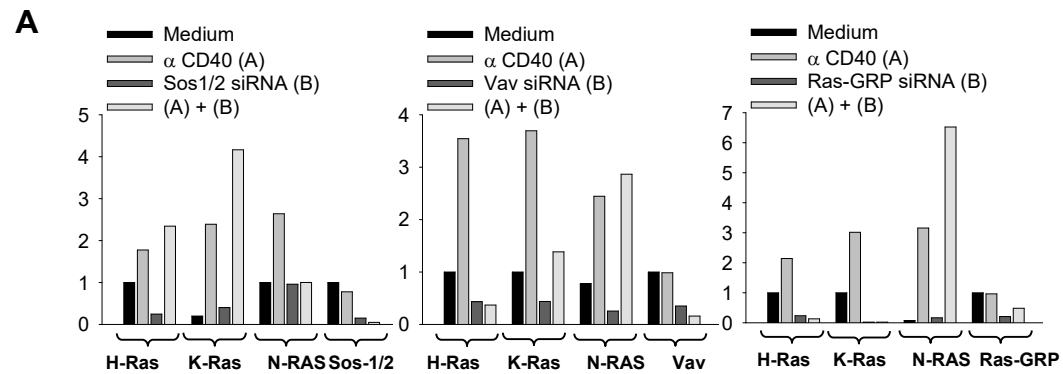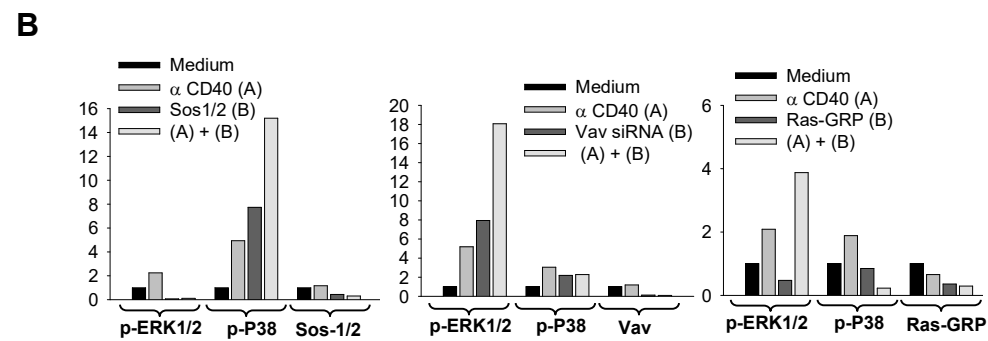

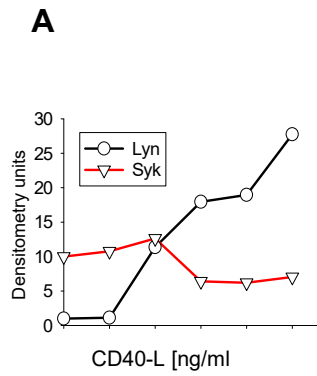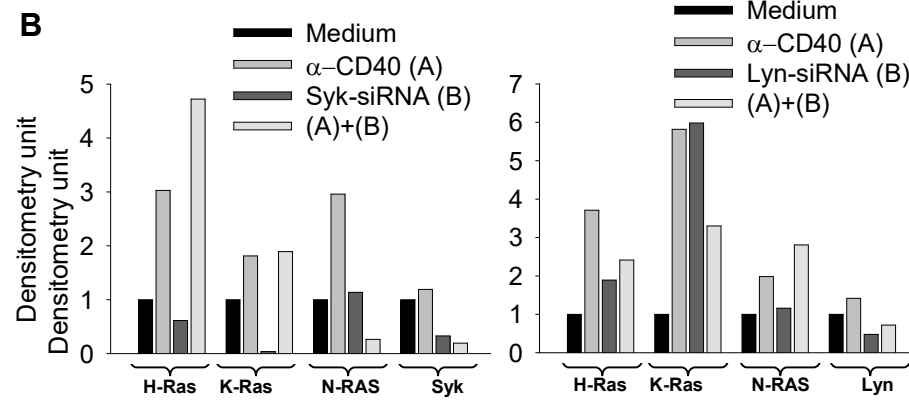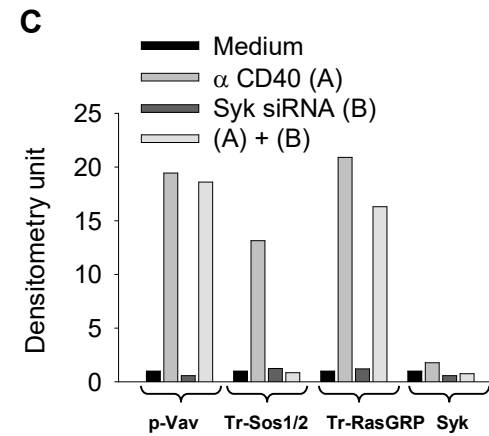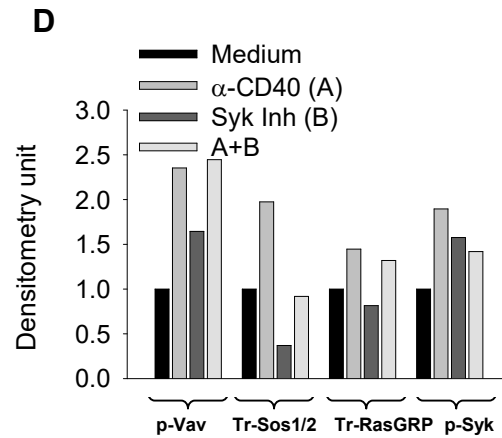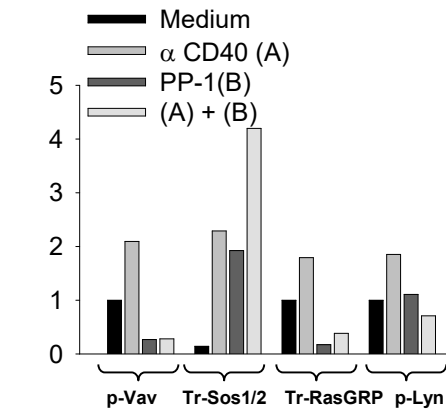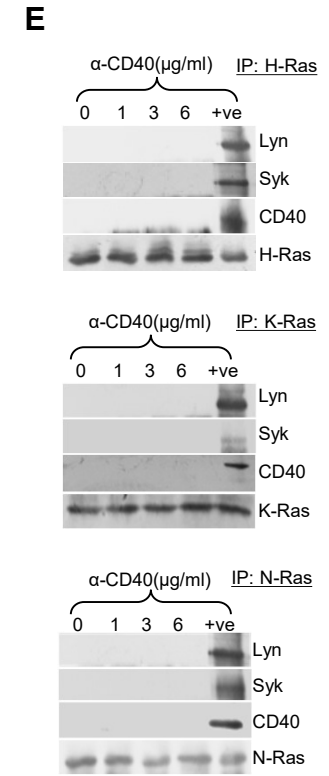

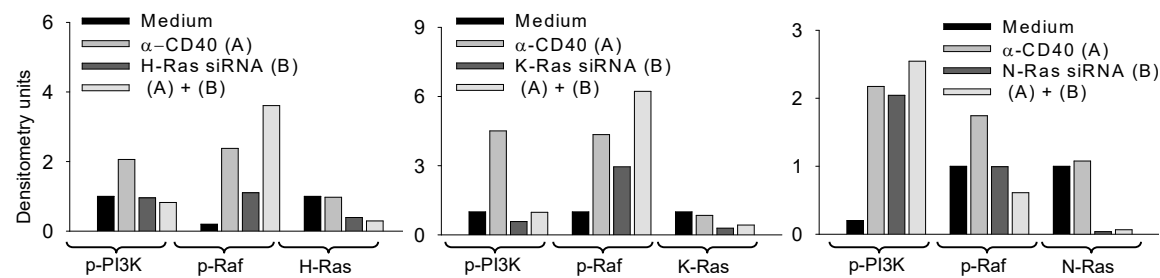

Supplement: Supplementary file 1 — Additional file 1. Figure S1. (A) Densitometry for immunoblot analysis for the silencing of Ras isoforms H, K, and N-Ras using specific siRNA. (B-D) Densitometry for immunoblots for phosphorylation of p38MAPK and ERK1/2 in P388D1 cells silenced for H (B), K (C), and N-Ras (D). Figure S2. (A) Densitometric analysis of the activation of H-Ras, K-Ras, and N-Ras on the silencing of Ras GEFs (Sos-1/2, Vav, and Ras-GRP) using GEF specific siRNA. (B) Densitometry of immunoblot analysis of phosphorylation of p38MAPK and ERK1/2 on silencing of Ras GEFs Sos-1/2, Vav and Ras-GRP. Figure S3. (A) Densitometric quantifications of the blots in Figure 5A. (B)Densitometric analyses of immunoblots of activated Ras isoforms in the lysates of untransfected or Syk or Lyn specific siRNA transfected, anti-CD40 antibody (3μg/ml) treated P388D1 cells, normalized to corresponding controls. (C) Densitometric analyses of immunoblots of translocated Sos-1/2 (Tr-Sos-1/2), translocated Ras-GRP (Tr-RasGRP), syk and lyn in the lysates of untreated or Syk siRNA or Lyn siRNA or anti-CD40 antibody (3μg/ml) treated P388D1 cells, normalized to corresponding controls. (D) Densitometric analyses of immunoblots of translocated Sos-1/2 (Tr-Sos-1/2), translocated Ras-GRP (Tr-Ras-GRP), phospho-lyn (p-lyn) and phospho-syk (p-syk) in the lysates of untreated or Syk inhibitor (Syk Inh, 3μM; Calbiochem, San Diego, CA) or PP-1 (340nM; BIOMOL International, PA) treated or anti-CD40 antibody (3μg/ml) treated macrophages, normalized to corresponding controls. (E) Co-immunoprecipitation of H-Ras, K-Ras and N-Ras at different doses of anti-CD40 to check for its association with Lyn, Syk and CD40. Figure S4. (A)Densitometry for effect of silencing of H-Ras, K-Ras, and N-Ras on the phosphorylation of PI3K and Raf. Text 1. Sequence and Structure Similarity Among Ras isoforms. Table S1. Sequence and structure similarity among Ras isoforms. Text 2. Comparative studies on symmetry of residue-residue interaction preferences, [file 12964_2019_497_MOESM1_ESM.zip › 12964_2019_497_MOESM1_ESM/Additional file Figure S1-4.pdf]
